# Surface-controlled spatially heterogeneous physical properties of a supramolecular gel with homogeneous chemical composition†

**DOI:** 10.1039/d1sc04671c

**Published:** 2021-10-11

**Authors:** Bin Yang, Marina Lledos, Riaz Akhtar, Giuseppe Ciccone, Long Jiang, Emanuele Russo, Sunil Rajput, Chunyu Jin, Maria Galini Faidra Angelereou, Thomas Arnold, Jonathan Rawle, Massimo Vassalli, Maria Marlow, Dave J. Adams, Mischa Zelzer

**Affiliations:** Department of Pharmacy, University of Nottingham Nottingham NG2 7RD UK mischa.zelzer@nottingham.ac.uk; Department of Mechanical, Materials and Aerospace Engineering, School of Engineering, University of Liverpool Liverpool L69 3GH UK; Centre for the Cellular Microenvironment, University of Glasgow Glasgow G12 8LT UK; Department of Chemical Engineering and Biotechnology, University of Cambridge Cambridge CB3 0AS UK; Diamond Light Source Ltd Harwell Science and Innovation Campus Didcot Oxfordshire OX11 0DE UK; European Spallation Source ERIC P. O. Box 176 SE-221 00 Lund Sweden; STFC, Rutherford Appleton Laboratory Chilton Didcot OX11 0QX UK; Department of Chemistry, University of Bath Claverton Down Bath BA2 7AY UK; School of Chemistry, University of Glasgow University Avenue Glasgow G12 8QQ UK

## Abstract

Controlling supramolecular self-assembly across multiple length scales to prepare gels with localised properties is challenging. Most strategies concentrate on fabricating gels with heterogeneous components, where localised properties are generated by the stimuli-responsive component. Here, as an alternative approach, we use a spiropyran-modified surface that can be patterned with light. We show that light-induced differences in surface chemistry can direct the bulk assembly of a low molecular weight gelator, 2-NapAV, meaning that mechanical gel properties can be controlled by the surface on which the gel is grown. Using grazing incidence X-ray diffraction and grazing incidence small angle X-ray scattering, we demonstrate that the origin of the different gel properties relates to differences in the architectures of the gels. This provides a new method to prepare a single domain (*i.e.*, chemically homogeneous) hydrogel with locally controlled (*i.e.*, mechanically heterogeneous) properties.

## Abstract 2

A mechanical pattern is created in a hydrogel film by pre-patterning the underlying surface chemistry. This allows spatial variation of the viscous component of the gel, controlling dissipative forces in the gel film without altering gel chemistry.

## Graphical abstract

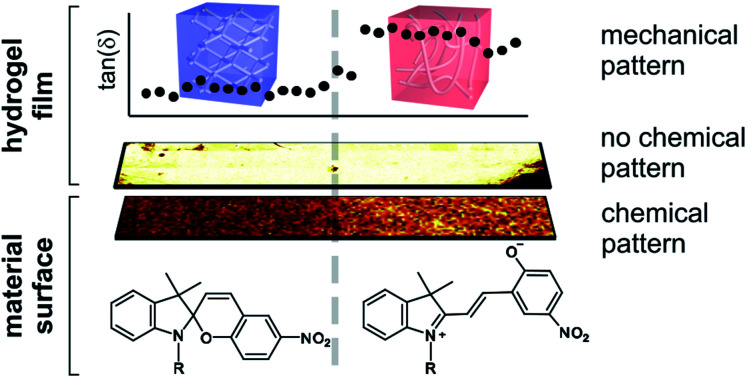

## Introduction

During the last decades, a growing body of evidence has highlighted the importance of the mechanical properties of the microenvironment in directing the development of living cells. Our understanding of this mechanism is evolving, with the recognition that dissipative (viscous) phenomena within the material, other than the conservative elastic component, are potently influencing the cellular fate.^1,2^ For example, by selectively attaching cell adhesion promoting molecules (collagen or fibronectin) to either the cross-linked (elastic component) or linear (viscous component) part of a polyacrylic acid based gels, Charrier *et al.* demonstrated that 3T3 fibroblast phenotype as well as the size of focal adhesions and paxicillin patches are affected by the ability of the polymer gel to undergo mechanical stress relaxation.^3^ Nevertheless, the details of this mechanism are still largely elusive, mostly due to the lack of suitable strategies to design substrates where the physical component (viscoelastic properties) can be controlled without altering the chemical composition^4^ but still retain the ability to spatially modulate the mechanical properties.

Spatial control over biomaterial properties provides the capability to influence the localised extracellular matrix (ECM) microenvironment to direct multistep biological processes (for example, cell differentiation or functional tissue regeneration),^5,6^ and/or control the release of pharmacologically-active molecules with differential kinetics and locations.^7^ Hydrogel patterning on a surface has been shown to be an effective strategy for aligning cells, which is critical in many important natural processes, such as the formation of functional vascular, muscular and neural tissues.^8^

The preparation of gels with localised properties across multiple length scales remains a challenge. Most strategies involve placing responsive heterogeneity within polymeric gels using specific chemical moieties.^9–11^ Polymer gels where specific patterns with different properties can be formed post-gelation can be prepared using polymers with tailored functionalities that interact with light.^9,12^ Multicomponent supramolecular gels have been prepared with spatially-resolved properties.^13,14^ Selectively irradiating sections of the gel through a mask leads to patterned, multicomponent networks. Recently, the addition of hyaluronic acid to an enzyme triggered peptide-based gelator was shown to result in different mechanical properties of the hydrogel.^15^

With these strategies, the mechanical properties of a supramolecular gel can be modulated by altering the chemical composition of the gel. However, this couples chemical and physical properties such that both aspects change if one needs to be adjusted. This is non-ideal in situations such as the design of cell culture supports where the same chemistry but different mechanical properties are required in the material to maintain biological compatibility and function of the chemical properties but match mechanical properties of materials with that of different tissues. There are currently no reports on preparing supramolecular hydrogels with controlled differential physical properties using a single component that would address this challenge.

Surfaces can play the role of a ‘seed layer’ that can initiate localised supramolecular hydrogelation.^16,17^ Several factors have been used to initiate supramolecular hydrogels exclusively near a surface,^18^ including electrostatic interactions,^19^ hydrophobicity/hydrophilicity,^20^ grafted gelators,^21^ pH,^22^ and enzymatic catalysis.^23–25^ Hydrogel patterning in 3D has been reported using urease immobilised on nanoparticles.^26^ Recently, surface topography has been used to fabricate a hydrogel with surface micro-features that can guide the orientation and shape of stem cells.^8^ Electrochemical-driven pH changes can also be used to prepare a spatially- and temporally-resolved multi-component gel at an electrode surface.^27^ The ability of surfaces to influence mechanical properties of supramolecular gels was demonstrated in principle;^20^ however, surface-initiated gel patterning has not yet been reported.

**Fig. 1:**
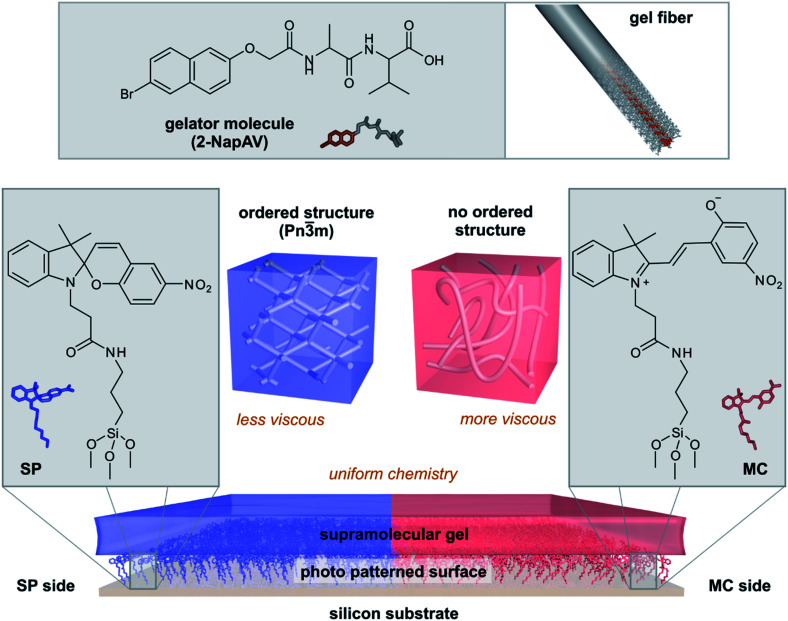
Surface-directed spatial control over supramolecular self-assembly. A surface modified with spiropyran is patterned with light to present both the open (MC) and closed (SP) forms of the molecule. Fibres formed from the gelator 2-NapAV arrange differently on the SP and MC sides of the patterned surface, forming a chemically homogenous but mechanically heterogeneous gel surface.

Here, we show that photo-patterned surfaces can be used to direct the bulk assembly of supramolecular hydrogels and thus produce gel films with different strengths of their viscous component while maintaining the same chemical composition (Fig. 1). The surface chemistry controls the self-assembly processes in the gels, leading to bulk gels with different properties. Surface-directed self-assembled structures can have a predictable impact on the mechanical properties of the final hydrogel. Our methodology allows hydrogels to be prepared with uniform chemistry but spatially-controlled variation in structure and mechanical properties.

## Results and discussion

### Preparation and switching of spiropyran surfaces

Spiropyran (SP) derivatives spontaneously isomerize to the open isomer merocyanine (MC) in response to UV radiation. The two isomers have very different physico-chemical properties in terms of charge, structure, p*K*_a_, and affinity to different chemical species.^28^ We modified a surface with a carboxyl-modified SP derivative (SP–COOH) (Fig. S1 and S2†), covalently immobilized to a silicon wafer surface *via* a (3-aminopropyl)trimethoxysilane (APTMS) linker (Fig. S4†).

Surface modification was confirmed using water contact angle (WCA) measurements (Fig. S4†) and Time-of-Flight Secondary Ion Mass Spectrometry (ToF-SIMS) (Fig. S5–S8†). The WCA of 4.7° ± 0.7° on cleaned silicon increased to 60.8° ± 4.8° and 90.2° ± 5.4° after reaction with APTMS and SP–COOH, respectively. ToF-SIMS spectra of these surfaces displayed a series of ions indicative of silicon (*m*/*z* 59.97, SiO_2_^−^), nitrogen containing compounds, *i.e.* APTMS and SP–COOH (*m*/*z* 26.0, CN^−^), and the spiropyran molecule (*m*/*z* 307.1, C_18_H_15_N_2_O_3_^−^). The reduction in SiO_2_^−^ and concomitant increase in CN^−^ normalized ion intensities after APTMS treatment indicates attachment of APTMS on the surface, while the appearance of the C_18_H_15_N_2_O_3_^−^ ion on the SP surface confirms immobilization of SP–COOH (Fig. S8†).

Switching from the SP to the MC form of SP–COOH on the surface by irradiation with UV light gave the sample a faint purple colour as previously reported.^28^ The transition also resulted in a change of the WCA from 90.2° ± 5.4° to 71.1° ± 6.0° (Fig. S4†), in line with previous data.^28^

In the ToF-SIMS spectra, the SP to MC transition results in a significant increase of the normalized ion intensities of the fragments at *m*/*z* = 137.0 (C_6_H_3_NO_3_^−^) and *m*/*z* = 150.0 (C_7_H_4_NO_3_^−^) (the latter is shown in detail in Fig. S8†). The opening of the chromene moiety after UV irradiation facilitates the generation of these fragments by cleavage at either the C–C or C=C bond between the two aromatic ring structures of merocyanine on the MC surface compared to the SP surface. The presence of a low-intensity signal of the C_7_H_4_NO_3_^−^ ion on the SP surface matches with the expectation that neither the SP nor the MC conversion is fully quantitative.^28^ Nonetheless, together the WCA and the ToF-SIMS data show that UV light induces the transition from SP to MC at the surface.

### Surface chemistry affects mechanical hydrogel properties

To investigate if the chemical differences between the SP and MC surfaces affect the properties of a self-assembled material, we prepared gels from a supramolecular gelator on both surfaces. 2-NapAV (Fig. 1) is a well-studied low molecular weight gelator with a pH-responsive sol–gel transition.^29^ At high pH, the terminal carboxylic acid is deprotonated and free-flowing solutions are formed. Gelation occurs when the pH is decreased, protonating the carboxylic acid group. To induce gelation, we use the hydrolysis of glucono-δ-lactone (GdL) to gluconic acid, which gives a slow, reproducible decrease in pH.^30^ This pH decrease is sufficiently slow so that there is time to transfer the solution to the surfaces before gelation has proceeded too far to present handling issues or introduce handling related artefacts in the gel structure.

The morphology of the fibres formed after gelation and the mechanical properties of the gel after drying were obtained from atomic force microscopy (AFM) measurements. Fig. S9a† shows the predominant presence of thick aggregates and twisted structures for the gel formed on the SP surface. In contrast, gels formed on the MC surface showed the presence of long fibre bundles with a width of about 50 nm (Fig. S9b†), which could result from fibre alignment induced by drying.

The relative mechanical properties (Young's modulus, *E*) of dried gels obtained on the two surfaces were measured with AFM-based nanoindentation (see ESI Section 6† for details). The influence of the substrate was ruled out by measuring and comparing the Young's modulus at different indentation depths before settling on a constant indentation depth for the experiment. The Young's moduli measured on both samples span a range of 10–750 MPa but show distinctly different distributions on the SP and the MC surfaces (Fig. S9c†). For gels formed on the SP surface, the peak modulus is just below 400 MPa. On the MC surface, the distribution appears bimodal, with a smaller population displaying a peak modulus similar to that observed on the SP sample and a larger population with a significantly lower peak modulus (approximately 50 MPa).

**Fig. 2:**
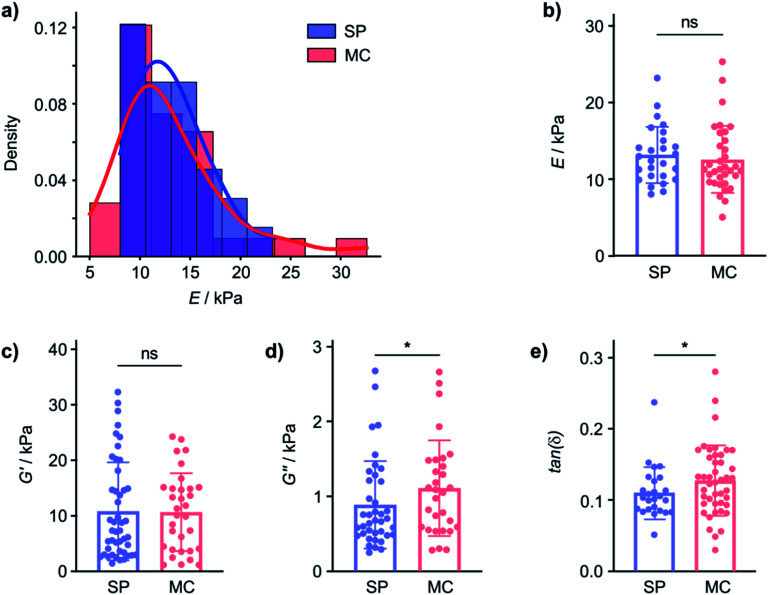
The same gelator forms gels with different mechanical properties on different surfaces. (a) Distribution of Young's modulus values (*E*) of hydrated 2-NapAV gels formed on an SP and an MC surface probed by nanoindentation (Optics11 system with a spherical tip radius of 22 μm). (b) The mean *E* of the hydrated gels on the SP and MC surfaces is not statistically significantly different (outliers removed using the ROUT method with *Q* = 1%, one tailed unpaired *t*-test, *p* = 0.2892, *n* = 2 for SP and *n* = 3 for MC). (c) The shear storage modulus (*G*^I^, converted from *E*^I^) of the gels prepared on SP and MC surfaces is not affected by surface chemistry (outliers removed using the ROUT method with *Q* = 1%, one tailed unpaired Mann–Whitney test, *p* = 0.2582, *n* = 1 for SP and *n* = 1 for MC), corroborating that hydrogels' elasticity is not modified by surface chemistry. (d) The shear loss modulus (*G*^II^, converted from *E*^II^) of the gels prepared on SP and MC surfaces is affected by surface chemistry (outliers removed using the ROUT method with *Q* = 1%, one tailed unpaired Mann–Whitney test, *p* = 0.0420, *n* = 1 for SP and *n* = 1 for MC). (e) The difference in *G*^II^ results in a statistically significant difference in tan(*δ*) = *G*^II^/*G*^I^ (outliers removed using the ROUT method with *Q* = 1%, one tailed unpaired Mann–Whitney test, *p* = 0.0454, *n* = 1 for SP and *n* = 1 for MC).

In contrast, the Young's modulus on hydrated samples, measured by nanoindentation (using the Optics11 system, see ESI, Section 7† for details), displayed similar distributions of *E* for the gel on both the SP and the MC surface and the mean *E* were not statistically significantly different (Fig. 2a and b). Even though the tip radius, and hence the contact area, is different for the AFM (*R* = 8 nm) and nanoindentation (*R* = 22 μm) measurements, and the exact values for *E* are not directly comparable, the distribution of the measured Young's moduli measured and the behaviour of the elastic and viscous components are clearly different in the dry and hydrated gel state. This indicates that while *E* is affected by the nature of the material surface when the gel is dry, the hydrated 2-NapAV gel does not display surface chemistry dependent *E*. The differences in mechanical properties in the wet and dry state of the 2-NapAV gel observed here are consistent with a previous study that reported different morphologies of this and other related gelators in the wet and dry state.^31^ While the present data demonstrates an intriguing surface-dependent influence on gel drying, to be relevant for subsequent applications of the hydrogel, differences in mechanical gel properties should, of course, pertain to the native, hydrated state of the gel.

To investigate if the SP and MC surfaces affect mechanical properties of gels that remain hydrated, we performed dynamic (oscillatory) nanoindentation experiments (using the Optics11 system) to probe both the storage (*G*′) and loss modulus (*G*′′) of the hydrated 2-NapAV gel on the SP and MC surfaces (Fig. 2c and d). On the hydrated gel, the *G*′ of the gel was not different on the two surfaces, indicating that elasticity of the hydrated gel is not affected by the substrate surface chemistry (Fig. 2a and b). In contrast, *G*′′ did show a statistically significant difference, indicating that the underlying surface chemistry affects the hydrogels' viscosity.

As mechanical characterisation of gel films is challenging and requires adaptation of the analytical approach to the nature of the sample, we will subsequently express the relationship between *G*′ and *G*′′ through the loss factor tan(*δ*). In fact, while *G*′ and *G*′′ are affected by the technique used to probe them, the loss factor, defined as the ratio between *G*′′ and *G*′, is independent of the magnitude of *G*′ and *G*′′ and independent of the contact area.^32^ Small tan(*δ*) values indicate a more elastic behaviour whereas large tan(*δ*) values are obtained from more viscous samples. The tan(*δ*) values from hydrated 2-NapAV gel on the SP and MC surfaces parametrise the differences in the mechanical properties of the gel films (Fig. 2e) in a manner that allows comparison across techniques, but it must be emphasised that, for the 2-NapAV gel films, these differences are associated with the viscous component (*G*′′) of the material.

### Patterned surface chemistry leads to chemically homogenous but mechanically heterogeneous hydrogel films

**Fig. 3:**
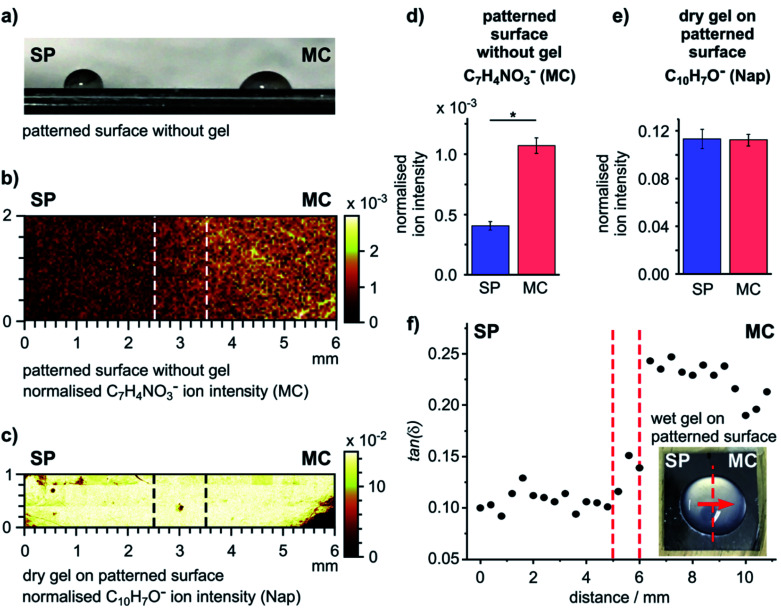
Photo-patterning results in surfaces and gel films with different characteristics. (a) Image of 10 μL water droplets on the two different sides of the patterned surface showing different wettability. (b) Prevalence of the MC state on the surface after photo-patterning indicated by the normalized intensity of the MC related ion C_7_H_4_NO_3_^−^ obtained by ToF-SIMS. (c) Chemical composition of the dry gel film formed on the patterned surface obtained by creating a ToF-SIMS image using a Nap related ion (*m*/*z* 143.0, C_10_H_7_O^−^). (d and e) Relative intensities of the MC (C_7_H_4_NO_3_^−^) and Nap (C_10_H_7_O^−^) related ions on the SP and MC sides of the patterned samples extracted from the data in (b) and (c). Values are reported as mean ± standard deviation; *n* = 4; the * indicates statistically significant difference (2 sample *t*-test, DF = 3, *p* = 2.576 × 10^−4^). (f) Oscillatory nanoindentation data for the patterned hydrogel in the wet state with a 100 μm spatial resolution. The inset shows an image of the hydrogel on the patterned surface. Nanoindentation was performed in the direction of the arrow across the centre of the hydrogel. Dashed lines indicate the interface region between the Vis (SP) and UV (MC) exposed parts of the sample.

As our surface design strategy allows spatial patterning of a single surface using light, we explored the ability to use a chemically patterned surface to create a single domain gel film with uniform chemical and heterogeneous mechanical properties. An SP surface was initially formed, and then irradiated selectively, with one half of the sample exposed to UV light (365 nm) and the other half exposed to white light (300–600 nm) simultaneously to drive predominant formation of the MC and SP forms of SP–COOH, respectively (Fig. S11†). The difference of the wettability on the two sides of the resulting surface (Fig. 3a) indicates successful surface photo-patterning.

The chemical patterning of the SP–COOH modified surface was visualized by ToF-SIMS imaging. The surface distribution of an ion indicative of SP–COOH (C_18_H_15_N_2_O_3_^−^), from both the SP and the MC forms, and an ion indicative of the MC form (C_7_H_4_NO_3_^−^) are shown in Fig. 3b and S12.† These images show a mostly uniform, but moderately patchy distribution of C_18_H_15_N_2_O_3_^−^ over the surface, indicating good coverage of SP–COOH. The C_7_H_4_NO_3_^−^ ion intensity was higher on the UV exposed side of the sample with a distinct transition between the two sides (Fig. 3b). The intensity difference of the C_7_H_4_NO_3_^−^ ion is statistically significant between the SP and MC sides (Fig. 3d), confirming spatially selective conversion of the surface into the SP and MC forms.

A gel was formed on this patterned surface in a large droplet (Fig. S13a, ESI†); this format reduces the influence of edge effects from the substrate on gel formation and provides the gel thickness and gel hydration required for the nanoindentation experiments performed in this work. The surface chemistry of the dry gel was analysed by ToF-SIMS, using the ion at *m*/*z* 143.0 (C_10_H_7_O^−^) that originates from the naphthyl structure of the gelator as marker for the gel. This ion distribution was homogenous over the interface (Fig. 3c) and showed no statistically significant difference between the two sides (Fig. 3e). This confirms that after formation of the gel on the patterned SP–COOH surface occurs on both sides of the sample and, importantly, that the chemistry of the gel film is identical across the whole sample.

To investigate how the surface patterning affected the mechanical properties of the gel, the micro-mechanical behaviour of the (hydrated) hydrogels (Fig. S13, ESI† for the sample format) was probed using oscillatory nanoindentation with a 100 μm flat punch indenter (using the KLA-Tencor system).^33^ This procedure can be used to study the mechanical properties of gels;^34,35^ it provides the shear storage modulus (*G*′) and the shear loss modulus (*G*′′) of each measurement point on the sample to allow us to determine tan(*δ*).

Nanoindentations were made across the centre of the patterned sample (inset, Fig. 3f), covering a distance of 10.8 mm with measurements taken at intervals of 400 μm. The loss factors as a function of distance are plotted in Fig. 3f (*G*′ and *G*′′ are plotted as a function of distance in Fig. S13b and c, ESI†). The hydrogel formed on the SP side had a statistically significantly smaller loss factor (tan(*δ*) = 0.107 ± 0.009) than the hydrogel on the MC side (tan(*δ*) = 0.225 ± 0.018) (2 sample *t*-test, DF = 24, *p* = 4.85 × 10^−17^). Similar to the data from gels on uniform surfaces (Fig. 2), this difference is due to the viscous component (*G*′′) of the gel (Fig. S13c, ESI†).

The same experiment was performed on non-patterned surfaces, conditioned towards either the SP or MC form (Fig. S14, ESI†). When comparing these full datasets obtained from the individual SP (tan(*δ*) = 0.173 ± 0.040) and MC (tan(*δ*) = 0.252 ± 0.020) surfaces, a significant difference in the tan(*δ*) values is observed (2 sample *t*-test, DF = 30, *p* = 7.14 × 10^−8^). The absolute value observed on the SP surface on patterned and non-patterned samples is not identical. This is ascribed to variations in sample properties inherent to gel formation as well as the possible influence of gel film thickness on the absolute value of the nanoindentation measurement. Nonetheless, the observed differences measured between the SP and MC surfaces are statistically significant.

To remove the possible influence of sample preparation parameters from the assessment of differences between patterned and non-patterned (control) surfaces, we separated the measurements from the individual SP and MC surfaces into two groups (left and right sides) for statistical comparison. No statistically significant difference was found between the two sides on either sample (2 sample *t*-test, *p* = 0.097 and 0.121 for SP and MC, respectively). These control measurements indicate that the relative differences observed on the patterned sample represents a real surface-controlled difference in mechanical properties of the gel film.

### Surface chemistry influences the tertiary structure of the supramolecular hydrogel

To investigate the origin of the differential mechanical properties of the hydrogels on the two different surface chemistries, we employed time-resolved grazing incidence X-ray diffraction (GID) and grazing incidence small-angle X-ray scattering (GISAXS) to elucidate the fibre structures and morphology in the hydrogel. Using both GID and GISAXS provided us with access to data for a wide length scale (1 to 125 Å) that enabled us to capture the primary (gelator–gelator distances), secondary (single fibre length scales) and tertiary (structure of the bulk system) structures of the hydrogels.

**Fig. 4:**
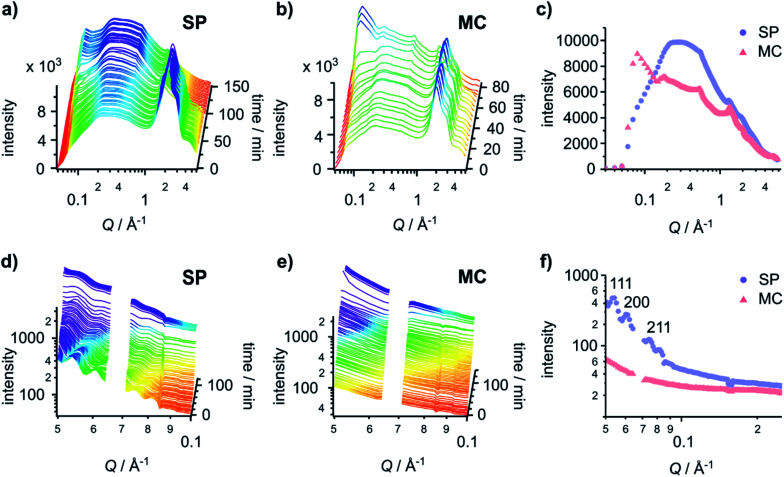
Gel fibres adopt structures with different packing orders on different surfaces. (Top) time-resolved GID patterns of hydrogels formed on (a) the SP surface and (b) the MC surface. (c) The final GID patterns of the hydrogel before it fully dries out on the SP surface (103 min) and the MC surface (76 min). (Bottom) time-resolved GISAXS patterns of the hydrogels formed on (d) the SP surface and (e) the MC surface. (f) The first GISAXS patterns (at 2 min) of the hydrogel on the SP surface and the MC surface. Reflection assignments are indicated in the graph. In (d)–(f), data between *Q* = 0.064 Å^−1^ and 0.071 Å^−1^ was omitted as it represents a gap in the detector.

Time-resolved GID data (Fig. 4a and b) provides information on the primary fibre structure. The peak and shoulder at 2.00 Å^−1^ and 2.86 Å^−1^ are water signals and are useful indicators for the presence of water in the sample. Gels on both the SP and MC surfaces are hydrated initially. The time required for the hydrogels to dry completely varies (103 min and 76 min on the SP and MC surfaces, respectively). This implies that the hydrogel formed on the SP surface has a higher ability to retain water between the fibres, likely due to a more lateral associated fibre network.

The GID patterns of the sample collected towards the end of the drying process display a much reduced water peak and are therefore more convenient for the identification of other peaks (Fig. 4c). Two peaks at 0.55 Å^−1^ (11.4 Å) and 1.34 Å^−1^ (4.7 Å) are present in the data for both surfaces, indicating that the primary structure of the gels is the same on both surfaces. The peak at 0.55 Å^−1^ corresponds to the distance between the centres of the naphthalene pairs along the fibre; it is slightly larger than the value collected previously from a dry gel.^30^ The 0.55 Å^−1^ peak is present as early as the first few patterns collected (Fig. 4a and b), indicating that the self-assembly process starts at a very early stage. The peak at 1.34 Å^−1^ is obscured by the water peaks and only appears when the gel is dry. This *d*-spacing corresponds well to the value^30^ ascribed to the hydrogen-bonded β-strand spacing.^36^ On 2D-GID images obtained on the SP surface (Fig. S15, ESI†), these two reflections are predominantly found in the direction of *q*_parallel_, indicating an in-plane ordering along the fibre axis on this surface.

Drying can promote fibre aggregation as the gelator molecules are predominantly uncharged when self-assembled.^31^ For the hydrogels formed on the SP surface, the reflection peaks at low *Q* are broader than those on the MC surface, indicating the presence of less well-defined structures on the SP surface. On the MC surface, hydrogels develop sharper reflection peaks at low *Q* during the drying process (Fig. 4c and S15, ESI†). The first three peaks at 0.09 Å^−1^, 0.18 Å^−1^ and 0.27 Å^−1^ with a *Q* ratio of 1 : 2 : 3 correspond to an ordered lamellar structure with a *d*-spacing of 69.8 Å. These three reflections in 2D-GID images (Fig. S15, ESI†) are aligned on the *q*_perpendicular_ axis, indicating an out-of-plane ordering of the bundles of dry fibres along the surface. This confirms that the drying process drives the alignment of the fibre bundles and aggregation into more ordered structures. However, there is no clear explanation for the observed lamellar phase of the aggregated fibres. These GID analyses suggest that the hydrogels formed on the SP surface have increased lateral association of fibres, which are then harder to align under the strain induced by the drying. This structure analysis corresponds well to the delayed drying time observed for SP surfaces and matches the observations made by AFM in Fig. 2, which indicate that on the MC surface the fibres are more aligned after drying.

To investigate the tertiary structure of the gels formed on the SP and MC surfaces, time-resolved GISAXS data were acquired (Fig. 4d and e). The large dip at 0.07 Å^−1^ is due to the gaps in the 2D detector. GISAXS patterns of hydrogels formed on the SP surface (Fig. 4d and f) showed three distinct peaks at 0.053 Å^−1^, 0.061 Å^−1^ and 0.075 Å^−1^. These three peaks were assigned to the (111), (200) and (211) reflections of a *Pn*3̄*m* phase with a unit cell of 205 Å. The peaks move to high *Q* values on drying, indicating a shrinkage of the unit cell by 1.8 nm due to water loss.

Compared to the gel on the SP surface, the absence of any obvious peaks from the gel on the MC surface indicates a lack of ordering of the fibres in the gel in both the wet and dry states (Fig. 4e). To give a qualitative indication of the size of the structures present in the gel on the MC surface, the initial pattern of the SAXS data was fitted with a Kratky–Porod flexible cylinder model with a polydisperse cross section and a uniform scattering length (see Fig. S15, S16 and Table S1, ESI,† for details).^37–39^ The fitting results indicate that the radius of the fibre is 77.9 ± 0.3 Å with a polydispersity of 0.802 ± 0.004 and a Kuhn length of 165.0 ± 15.3 Å. While this analysis is not truly quantitative, it does give a fitted fibre diameter that is similar to the reported value obtained from small-angle neutron scattering (SANS) data fitting.^40^ This procedure could not be carried out for the gel on the SP surface as the model only applies to systems that do not include a structure factor. The presence of peaks in the first patterns on the SP surface indicates that the gel includes significant structural interactions.

The appearance of lamellar structures obtained from the time-resolved GID data during the drying process indicates a more lateral associated gel network on the SP surface as compared to the MC surface. The GID data show the same primary structure on both SP and MC surfaces, while GISAXS data confirm a very ordered tertiary gel network structure formed on the SP surface and an amorphous tertiary gel network on the MC surface. The *Pn*3̄*m* packing order has been observed in lipid-like^41^ and polymer based^42^ self-assembled structures, but *Pn*3̄*m* ordered tertiary gel packing has not been reported previously for self-assembling peptide amphiphiles. Hence, our data suggest that the difference in the mechanical properties of the gels is not driven by the primary self-assembled structure of the fibre itself, but by variations in fibre secondary or tertiary structure that are influenced by the self-assembly processes initiated on the surface.

The properties of the gels formed from this class of gelator are determined by the kinetics of the pH change.^30^ However, various surface properties such as polarity, charge, topography and molecular structures can influence self-assembly.^43^ The SP and MC surfaces used here display a range of significant differences in their properties. On the SP surface, the spiropyran is rigid and non-planar. However, upon irradiation with UV light, the conformation changes to a more flexible structure with two planar ring systems on the MC surface. This structural change is accompanied by differences in polarity, charge and acidity. We hypothesize that these differences directly translate into how the growing fibres of 2-NapAV can interact with and template from the surface. While highly ordered and more laterally associated, the individual fibres of the hydrogel formed on the SP surface appear shorter than on the MC surface. Despite being more disordered, the longer fibres of hydrogels on the MC surface presumably lead to a higher persistence length compared to fibres on the SP surface, resulting in a higher viscous component (*G*′′) of gels on the MC surface. The association between increasing viscosity and increasing rigidity due to polymer chain stiffness is known in the literature.^44^ In addition to fibre persistence length, the difference in water retention in hydrogels on the SP and MC surfaces may lead to differences in gel fibre concentrations due to water sequestration that could contribute to the observed increase in the viscous component of the MC surface. The mechanical properties of another class of gelators, an amphiphilic nucleobase derivative,^45^ have also been shown to be affected by surface properties,^20^ suggesting that chemical patterning of surfaces could find broader application to control mechanical gel properties.

## Conclusions

Controlling the supramolecular self-assembly across multiple length scales to prepare hydrogels with spatially localised properties is challenging. Normally, this is achieved by using spatially-resolved multicomponent hydrogels, where spatial differences in physical properties are accomplished by changes in the chemical composition of the material. Chemical modification of the material to alter physical properties is not always ideal as it increases material complexity and reduces the ability to adopt material properties independent of each other.

We have shown that different surface chemistries can be used to direct the self-assembly of gelator molecules in a manner that leads to differences in the bulk gel physical properties. This method can be used to form a hydrogel with spatially-resolved structure and viscous properties but uniform chemical composition. This represents a significant step forward from the current state of the art as it allows modulation of viscous properties of supramolecular gels without altering the chemical composition of the gel. The observed differences in the secondary and tertiary structures could be related to several processes such as differences in the gelator nucleation, self-assembly process and kinetics caused by differences in the physicochemical properties such as molecular structure, charge and acidity of SP and its isomer MC. The precise underlying mechanism remains uncertain and would have to be the subject of future investigations.

This methodology opens a possibility of preparing hydrogels with uniform chemistry throughout, but spatially controlled variation in self-assembled structure and viscous properties. The ability to control the surface chemistry by photo-patterning provides a convenient route to adapt the method to more complex patterns or applications. This approach should allow gels to be prepared where differences in viscous properties do not require a difference in the absolute chemistry, which should be of great utility for applications in cell growth for example. While the differences in *G*′′ observed for the present 2-NapAV system are relatively small in the hydrated state (tens to hundreds Pa), they are statistically significant and in the same order of magnitude as some critical biological dissipative viscoelastic processes.^1^ These differences are therefore of potential interest to biological applications. Nonetheless, it will be interesting to explore ways to increase the range of differences in viscoelastic properties that are accessible in future.

## Experimental

Detailed procedures for the sample preparation and material analysis, including synthetic procedures, WCA, AFM, ToF-SIMS, nanoindentation, GID and GISAXS are available in the ESI.†

### Surface patterning

A patterned surface where one half displayed the closed SP form and the other the open MC form of the spiropyran derivative was obtained by selectively irradiating half of the modified surface with white light (300–600 nm, 3 W) and the other half with UV light (365 nm, 8 W, 0.458 mW cm^−2^ at sample position) simultaneously for 12 hours to ensure maximum conversion into the respective form. The surfaces were used immediately after irradiation.

### Hydrogel preparation

The gelator, 2-NapAV, was synthesised as described previously.^30^ To prepare the hydrogel, 2-NapAV (2.5 mg) was suspended in deionized water (0.5 mL). An equimolar quantity of NaOH (0.1 M) was added and the mixture was gently stirred for two hours until a clear solution was formed. The pH of this solution was 10.7. Then, glucono-δ-lactone (GdL, 2.32 mg) was added and the solution swirled. 350 μL of the solution was applied to a modified surface and left overnight in a sealed Petri-dish to ensure complete gelation. The remainder of the solution was kept in the sample tube as control to ensure that gelation had occurred. The final pH of this gel was 4.2.

### Nanoindentation (homogenous gels)

Nanoindentation experiments to probe the Young's modulus *E* of wet gels on homogenous surface (Fig. 2 and S10, ESI†) were performed using a nanoindentation device (Chiaro, Optics 11) mounted on top of an inverted phase contrast microscope (Evos XL Core, Thermo Fisher). Measurements were performed at room temperature on hydrated gels.

Static measurements were performed by acquiring a set of single force–displacement (*F*–*z*) curves for each sample, at a constant speed of 2 μm s^−1^ over a vertical range of 10 μm, changing the position on the sample at every indentation by laterally displacing the tip ∼250 μm.

For dynamic nanoindentation experiments on homogeneous gels (Fig. 2 and S10, ESI†), for each sample, a set of curves was acquired by performing two matrix scans in different regions of the gel. Each matrix scan consisted of 25 indentations, with a lateral step in *x* and *y* of 50 μm. Indentations consisted of stress relaxation experiments in dynamic mode, where a step indentation of 2000 μm with superimposed sinusoidal oscillations of amplitude 5 nm at 1, 2, 4 and 10 Hz was used as the input signal. The relaxing force was recorded over the time of the indentation, which was set to approximately 20 s (see Fig. S10, ESI†).

This method directly measures compression elastic moduli *E*′ and *E*′′ but to simplify the comparison with oscillatory nanoindentation and other rheological measurements in the literature, we treated the material as a linear incompressible solid and converted the values to *G*′ and *G*′′.

The cantilever selected for all the experiments (static and dynamic) had an elastic constant *k* of 0.34 N m^−1^ and held a spherical tip of radius (*R*) 22 μm in radius. Full methodological details are given in the ESI.†

### Oscillatory nanoindentation (patterned gels)

Oscillatory nanoindentation was performed to determine the complex shear modulus of gel films on patterned surfaces (Fig. 3, S13 and S14, ESI†) using a KLA-Tencor Nanoindenter G200 with an ultra-low load DCM-II Head (Milpitas, California). Measurements were conducted with a 100 μm flat punch indenter (Synton-MDP Ltd., Nidau, Switzerland) at room temperature (25 °C) at 110 Hz with a pre-compression of 5 μm. Full methodological details including the underlying theory for this method have been reported elsewhere.^33^ 350 μL of the solution of 2-NapAV and GdL were placed on the surface to form gel films with a thickness of approximately 3 mm. 30 indentations at 400 μm intervals were made across the centre of the sample, covering a distance of about 10.8 mm. *G*′, *G*′′ and the loss factor tan(*δ*) (*i.e.*, the ratio of *G*′′/*G*′) were calculated for each indentation. After each indentation, the tip was cleaned by indenting a piece of double-sided Scotch tape mounted on an adjacent sample puck before returning to the gel sample.

### Grazing incidence X-ray diffraction (GID) and grazing incidence small-angle X-ray scattering (GISAXS)

The sample was prepared using the method described above on a SP–COOH modified silicon wafer surface. GID and GISAXS experiments were performed on the I07 beamline, Diamond Light Source, Didcot, UK using a Pilatus 2M detector.^46^ Time-resolved GID experiments were started immediately after spreading of the solution on the surface occurred (without sample realignment) using X-rays with an energy of 18 keV and a wavelength of 0.6888 Å to achieve a *Q*-range of 0.05–5.8 Å^−1^. The sample-detector distance was 30 cm. For the time-resolved GISAXS experiment, patterns with a *Q*-range of 0.027–0.6 Å^−1^ were obtained using an X-ray energy of 14.5 keV and a wavelength of 0.8551 Å. The sample-detector distance was 3 m. In both cases, the incident angle was 0.08°, just below the critical angle for the substrate and ensuring surface sensitivity. The data was analysed using IGOR PRO v6.37 and the SANS analysis package v4.20 (Package 7.0).

## Data availability

Additional data is available in the ESI† and raw data is available from the University of Nottingham data repository at the persistent identifier DOI: 10.17639/nott.7151.

## Author contributions

B. Y., M. Z., D. J. A. and M. M. conceptualised the project. B. Y., M. L., M. M., D. J. A., M. Z. (all parts), R. A. and G. C. (nanoindentation), E. R., S. R. (spiropyran synthesis), M. G. F. A. and T. A., J. R. (X-ray scattering), developed methodologies. B. Y., M. L. (all parts), R. A. (nanoindentation), L. J. (ToF-SIMS), T. A. and J. R. (X-ray scattering) contributed to the investigation. Resources (spiropyran derivatives, gelator) were provided by E. R., S. R. and D. J. A. Formal data analysis was carried out by B. Y., M. L., R. A., L. J., E. R., S. R., C. J., G. C. and M. Z. B. Y., M. L., T. A., J. R. G. C. and M. Z. were responsible for data curation. M. Z., D. J. A., M. M. and M. V. supervised the project and M. Z. was responsible for project administration. Visualisation and writing of the original draft was performed by B. Y., M. L., G. C. and M. Z. All other authors contributed to reviewing and editing the draft. Funding acquisition was done by M. Z., M. M., D. J. A. (Leverhulme Trust) and B. Y., M. G. F. A. and M. Z. (Diamond beamtime).

## Conflicts of interest

There are no conflicts to declare.

## Supplementary Material

SC-012-D1SC04671C-s001
