# Supplementary material for: Surface-controlled spatially heterogeneous physical properties of a supramolecular gel with homogeneous chemical composition
Source: Chem Sci. 2021 Oct 11;12(42):14260–9. doi: 10.1039/d1sc04671c (PMC8565383; doi:10.1039/d1sc04671c)
Supplement: SC-012-D1SC04671C-s001 [file SC-012-D1SC04671C-s001.pdf]

Electronic Supplementary Material (ESI) for Chemical Science.
This journal is © The Royal Society of Chemistry 2021

Surface-controlled spatially heterogeneous physical properties of a supramolecular gel with homogeneous chemical composition

Supplementary Information

Bin Yang,[1] Marina Lledos,[1] Riaz Akhtar,[2] Giuseppe Ciccone,[3] Long Jiang,[1] Emanuele Russo,[1] Sunil Rajput,[1] Chunyu Jin,[4] Maria Galini Faidra Angelerou,[1] Thomas Arnold,[5,6,7,8] Jonathan Rawle,[5] Massimo Vassalli,[3] Maria Marlow,[1] Dave J. Adams[9] and Mischa Zelzer[1,*]

[1] Department of Pharmacy, University of Nottingham, Nottingham, NG2 7RD, United Kingdom
[2] Department of Mechanical, Materials and Aerospace Engineering, School of Engineering, University of Liverpool, Liverpool L69 3GH, United Kingdom
[3] Centre for the Cellular Microenvironment, University of Glasgow, Glasgow, G12 8LT, United Kingdom
[4] Department of Chemical Engineering and Biotechnology, University of Cambridge, Cambridge, CB3 0AS, United Kingdom
[5] I07 Diamond Light Source Ltd, Harwell Science and Innovation Campus, Didcot, Oxfordshire, OX11 0DE, United Kingdom
[6] European Spallation Source ERIC, P.O Box 176, SE-221 00 Lund, Sweden
[7] STFC, Rutherford Appleton Laboratory, Chilton Didcot, OX11 0QX , UK
[8] Department of Chemistry, University of Bath, Claverton Down, Bath BA2 7AY, UK
[9] School of Chemistry, University of Glasgow, University Avenue, Glasgow, G12 8QQ, United Kingdom

*Corresponding author: mischa.zelzer@nottingham.ac.uk

Contents

1. Materials 3
2. Synthetic procedures 3
   2.1. Spiropyran derivative 3
   2.2. Gelators 5
3. Surface modification 5
   3.1. Preparation of spiropyran-modified surfaces 5
4. Surface characterisation 6
   4.1. Water contact angle 6
   4.2. ToF-SIMS 6
5. Photo-induced surface switching 9
6. Morphological and mechanical properties of dry gel films 10
7. Mechanical properties of uniform hydrated gel films 11
   7.1. Static Nanoindentation Experiments 11
   7.2. Oscillatory Nanoindentation Experiments 13
8. Characterisation of patterned surfaces 14
   8.1. Preparation of patterned surfaces 14
   8.2. Chemical characterisation of surfaces by ToF-SIMS 14
   8.3. Mechanical characterisation of patterned hydrated gels by oscillatory nanoindentation 16
9. Gel fibre structure 18
   9.1. Grazing-incidence X-ray diffraction (GID) 18
   9.2. Grazing-incidence small-angle X-ray scattering (GISAXS) 20
10. Replicates and statistical analysis 21
11. References 23

1. Materials

3-Iodopropionic acid (95%, I10457), piperidine (99%, 104094), *N*,*N*'-Diisopropylcarbodiimide (≥98%, 38370) and anhydrous *N*,*N*-Dimethylformamide (99.8%, 227056) were purchased from Sigma Aldrich. 2,3,3-Trimethylindolenine (98%, 140161000) and 2-hydroxy-5-nitrobenzaldehyde (98%, 416180250) were obtained from Acros Organics. Chloroform (HPLC grade, C/4966/17), methyl ethyl ketone (laboratory reagent grade, E/1450/17), hydrogen peroxide (>30% w/v, H/1750/17), methanol (HLPC grade, M/4056/17), toluene (analytical reagent grade, T/2300/17) and sulphuric acid (>95%, S/9160/PB17) were acquired from Fisher. (3-Aminopropyl)trimethoxysilane (97%, A1128422) was purchased from Alfa Aesar. Silicon wafers were reclaim grade P(Boron), 4 inch diameter, 425-550 µm thick, P(Boron), 0-100 ohm cm, single side polished obtained from PI-KEM Limited. The wafers were cut to size with a diamond pen.

2. Synthetic procedures

2.1. Spiropyran derivative

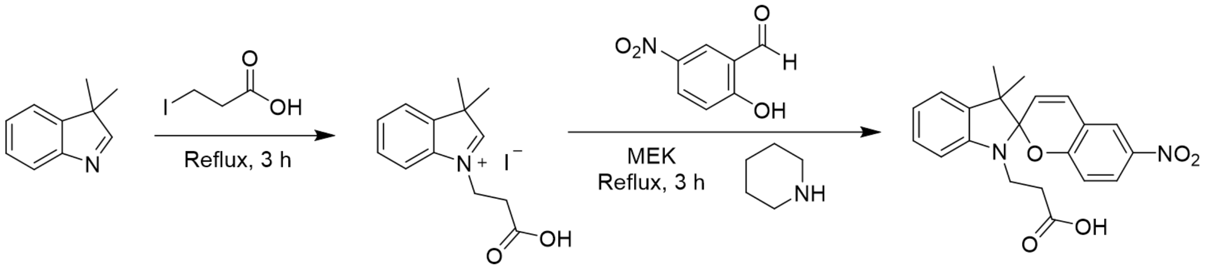

(1) **(2)**

Figure S1. Synthesis of SP-COOH (**2**) via a two-step reaction.

1-(2-Carboxyethyl)-2,3,3-trimethyl-3H-indol-1-ium iodide (1). Synthesis of (**1**) was performed according to a previously reported procedure (Figure S1).[1] 2,3,3-Trimethyl-3*H*-indole (4 g, 25 mmol, 1 eq) and 3-Iodopropanoic acid (5.4 g, 27 mmol, 1.1 eq) were mixed in a round bottom flask and the reaction mixture was stirred under reflux for 3 hours. After cooling to room temperature, the obtained red solid was dissolved in water (100 mL) and stirred overnight. The solution was washed with chloroform (5 x 50 ml). The yellow water phase was collected and the product obtained via lyophilisation as light-brown crystals of (**1**) (yield: 95%). The product was used for the next reaction step without further purification. The ^{1}H NMR spectrum of (**1**) is shown in Figure S2.
^{1}H NMR (300 MHz, DMSO-d_6) / ppm: 12.75 (bs, 1H, COOH), 7.98 (m, 1H, H_e), 7.84 (dt, J = 7.6 Hz, J = 3.5 Hz, 1H, H_d), 7.62 (m, 2H, H_b, H_c), 4.64 (t, J = 7.0 Hz, 2H, H_f), 2.97 (t, J = 7.0 Hz, 2H, H_g), 2.86 (s, 3H, H_i), 1.52 (s, 6H, H_a).

3-(3',3'-Dimethyl-6-nitrospiro[chromene-2,2'-indolin]-1'-yl)propanoic acid (SP-COOH) **(2).** Spiropyran (**2**) was prepared according to a literature procedure.[1] (**1**) (4.5 g, 12.5 mmol, 1 eq) was dissolved in methyl ethyl ketone (MEK) (100 mL) in a round-bottom flask covered with aluminium foil. Piperidine (1.3 mL, 13.2 mmol, 1.1 eq) and 2-hydroxy-5-nitrobenzaldehyde (2.1 g, 12.5 mmol, 1 eq) were added and the red reaction mixture was stirred under reflux for 3 hours. Then, the reaction mixture was cooled to room temperature and stored for 12 hours at 0 °C. The precipitate was filtered and washed with methanol (3 x 50 mL) to obtain (**2**) as a yellow powder (yield: 60%). The ^{1}H NMR spectrum of (**2**) is shown in Figure S2.

^{1}H NMR (300 MHz, DMSO-d_6) / ppm: 12.24 (s, 1H, COOH), 8.23 (d, J = 2.3 Hz, 1H, H_k), 8.00 (dd, J = 8.9 Hz, J = 2.4 Hz, 1H, H_l), 7.21 (d, J = 10.4 Hz, 1H, H_m), 7.13 (m, 2H, H_b, H_d), 6.87 (d, J = 9.0 Hz, 1H, H_j), 6.80 (t, J = 7.3 Hz, 1H, H_c), 6.66 (d, J = 7.8 Hz, 1H, H_e), 6.00 (d, J = 10.4 Hz, 1H, H_i), 3.5-3.3 (m, 2H, H_f), 2.62 (m, 2H, H_g), 1.2 (s, 3H, $H_{a'}$), 1.09 (s, 3H, H_a).

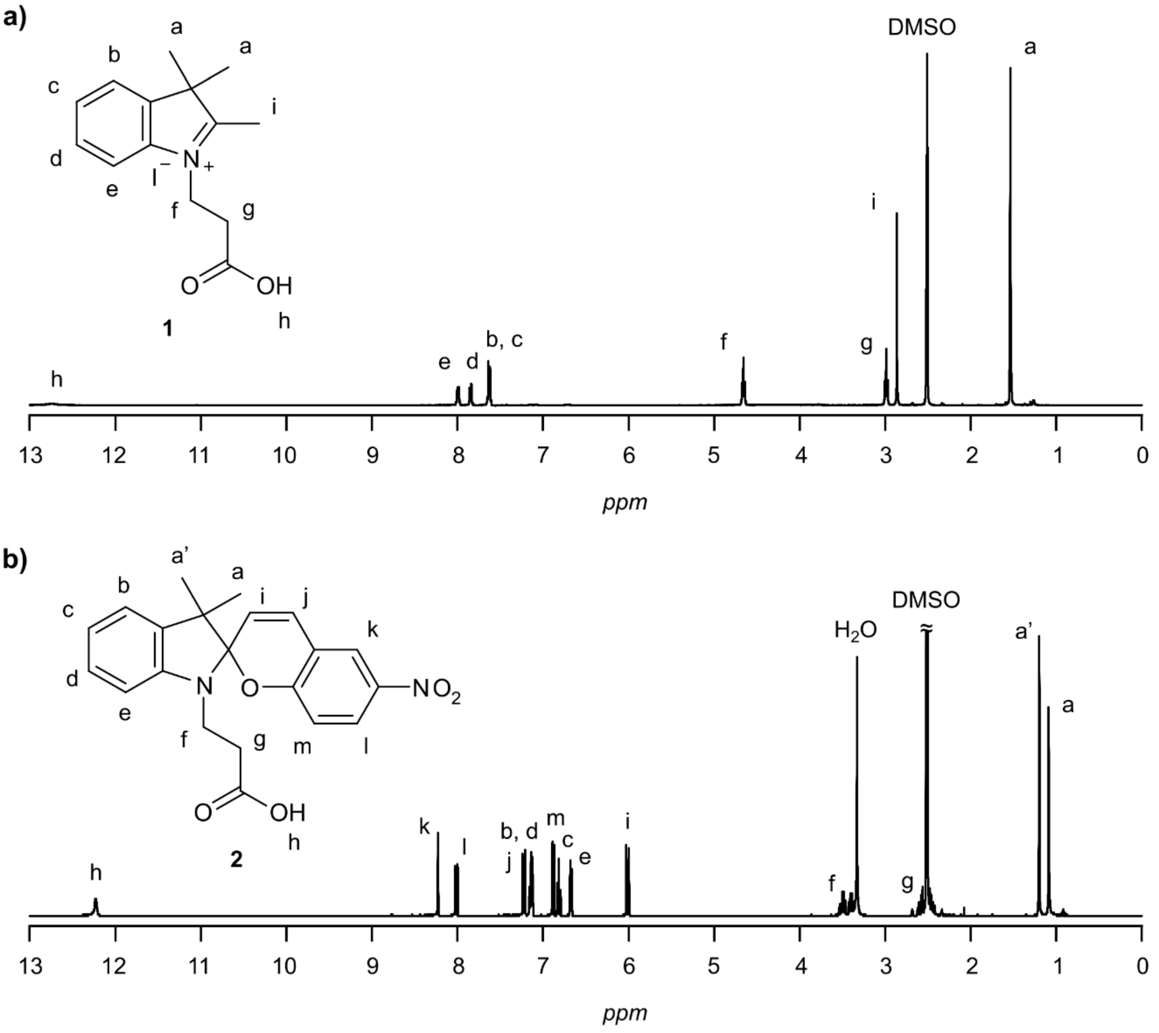

Figure S2. ^{1}H NMR spectra of (**1**) and (**2**) in DMSO-d_6 recorded at 300 MHz.

2.2. Gelators

The gelator used in this work, 2-NapAV (2-(2-(2-6-Bromonaphthalen-2-yloxy) acetamido) propanamido)-3-methylbutanoic acid, Figure S3), was synthesised following a method previously reported by Chen *et al*.[2]

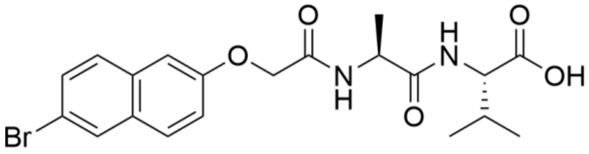

Figure S3. Structure of 2-NapAV.

3. Surface modification

3.1. Preparation of spiropyran-modified surfaces

To create spiropyran-modified surfaces, clean silicon wafers were modified with amine functionalities through which (**2**) was immobilised on the surface (Figure S4). The samples were placed in petri dishes and cleaned by sequentially sonicating them for five minutes in methanol, acetone and isopropanol. The substrates were dried using compressed air. Sample cleaning and exposure of hydroxyl groups on the surfaces was accomplished via piranha cleaning. Piranha solution (20 mL) (*Caution: Piranha solutions reacts violently with organic materials*) was added to a glass petri dish containing the samples and left to react for 40 minutes. The samples were rinsed and sonicated in Millipore ultrapure water for ten minutes, then dried using compressed air.

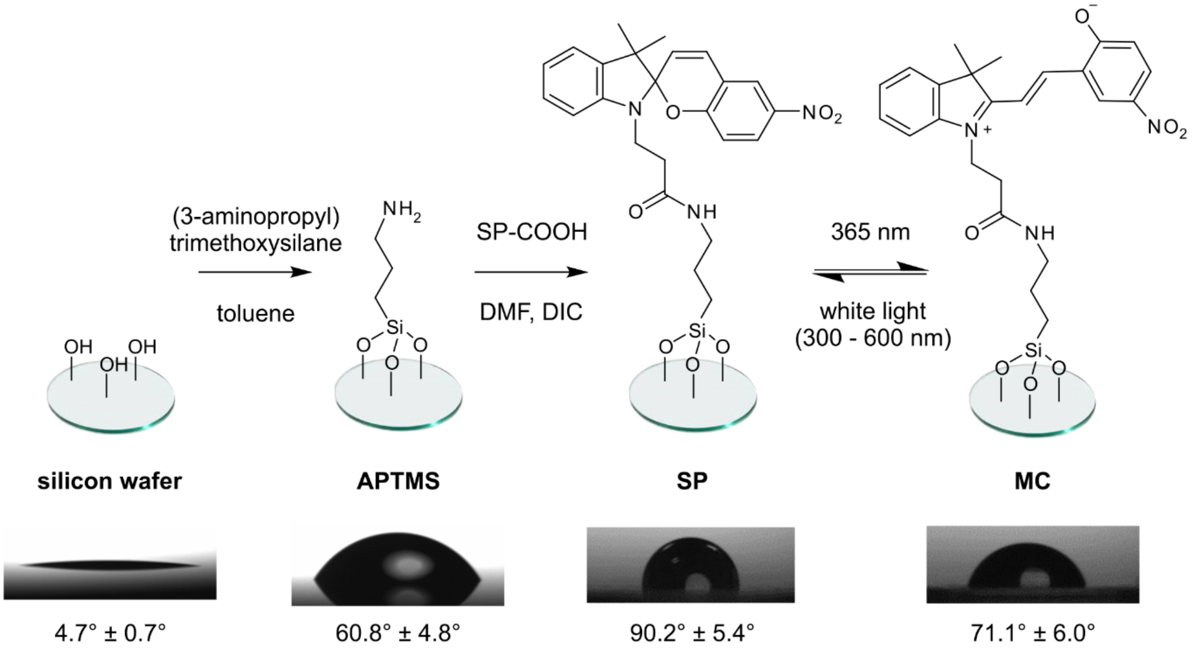

Figure S4. Substrate functionalisation with SP-COOH (**2**) via 3-aminopropyl)trimethoxysilane (APTMS) followed by diisopropylcarbodiimide (DIC) coupling of (**2**) onto the surface. The resulting SP-COOH modified surface (SP) was converted to the merocyanine form (MC) upon exposure to 365 nm light. Below the schematic, representative water droplets and measured water contact angles on the surfaces show the change in hydrophobicity of the surface at each modification step (n = 5, 6, 6, and 4 for the silicon wafer, APTMS, SP and MC surfaces, respectively).

Amine functionalisation of the samples was accomplished via silanisation of the slides with (3-aminopropyl)trimethoxysilane (APTMS). The slides were placed in a glass petri dish and a 1% solution of APTMS in toluene (20 mL) was added. The samples were incubated at room temperature for one hour, followed by washing and sonicating sequentially with toluene, acetone and Millipore ultrapure water for five minutes each, and drying under compressed air.

The final step of the modification involved the addition of SP-COOH to APTMS modified surfaces. A solution of SP-COOH in anhydrous DMF (95 mM, 13.9 mL) and diisopropylcarbodiimide (DIC) (410 µL) were added to a glass petri dish containing the APTMS functionalised slides. This was left at room temperature on a Heidolph Rotamax 120 Orbital Shaker at 20 rpm for 16 hours. After incubation, the slides were sequentially washed in DMF, acetone and water for five minutes each, and then dried using compressed air. The slides were stored in the dark in a desiccator under vacuum.

4. Surface characterisation

4.1. Water contact angle

At each step of the surface modification, water contact angle (WCA) measurements were used to monitor changes in surface properties. The WCA was determined with a KSV Cam200 Optical Contact Angle Meter. The Cam200 was set up to record 20 frames at a speed of one frame per second for each droplet. The WCA of the droplet in each frame was calculated using the circle fitting method. As the droplets are not immediately in equilibrium after deposition on the surface, values from the first two frames were discarded to reduce the likelihood of the data to be affected by WCAs measure in a non-equilibrated state. The WCA for the remaining frames were used to extrapolate the WCA at time-point zero, which was used as the WCA value for one measurement. The reported WCA measurements are presented as average ± standard deviation (SD). n = 5, 6, 6, and 4 for the silicon wafer, APTMS, SP and MC surfaces, respectively.

The WCA angles increased at each modification step from 4.7° ± 0.7°, to 60.8° ± 4.8° and 90.2° ± 5.4° for silicon, APTMS and SP, respectively (Figure S4). The increasing hydrophobicity indicates changes to the surface chemistry at each modification step.

4.2. ToF-SIMS

To analyse the chemical composition of the surfaces, ToF-SIMS was carried out with a ToF-SIMS IV instrument (ION-TOF GmbH, Münster, Germany) using a 25 keV Bi_3^+ primary ion source with a target current of approximately 1.0 pA. Charge compensation was applied via a low energy (20 eV) electron floodgun. SIMS data were acquired in negative polarity over regions of 500 µm × 500 µm at 256 × 256 pixel resolution for 20 scans or over large areas of 2 mm x 6 mm (or 1 mm x 6 mm) at 100 pixels / mm for1 scan. The data was analysed with SurfaceLab 6 (ION-TOF GmbH). Negative ion mass spectra were calibrated to CH^- (*m/z* 13), CH_2^- (*m/z* 14), C_3H^- (*m/z* 37) and C_4H^- (*m/z* 49).

Full ToF-SIMS spectra of negative ions detected on the samples are shown in Figures S5 and S6. Ions indicative for the respective samples were identified in these spectra are *m/z* 59.97 (SiO_2^-, silicon), *m/z* 26.0 (CN^-, APTMS, SP and MC), *m/z* 307.1 ($C_{18}H_{15}N_2O_3^-$, SP), *m/z* 137 ($C_6H_3NO_3^-$, MC) and *m/z* 150 ($C_7H_4NO_3^-$, MC). The assigned structures for the SP and MC related ions are shown in Figure S7 and the corresponding signals in the ToF-SIMS spectra are shown in more detail in Figure S8.

The modification of silicon with APTMS showed an increase in CN^- ion intensity and a decrease in SiO_2^- ion intensity, confirming that the surface was modified with a nitrogen containing compound (APTMS). The appearance of the molecular ion of the full spiropyran molecule ($C_{18}H_{15}N_2O_3^-$) on the SP surface indicates attachment of SP-COOH on the surface. CN^- ion intensity remains at similar levels because CN^- is a generic indicator of nitrogen containing material on the surface and SP-COOH also contains nitrogen.

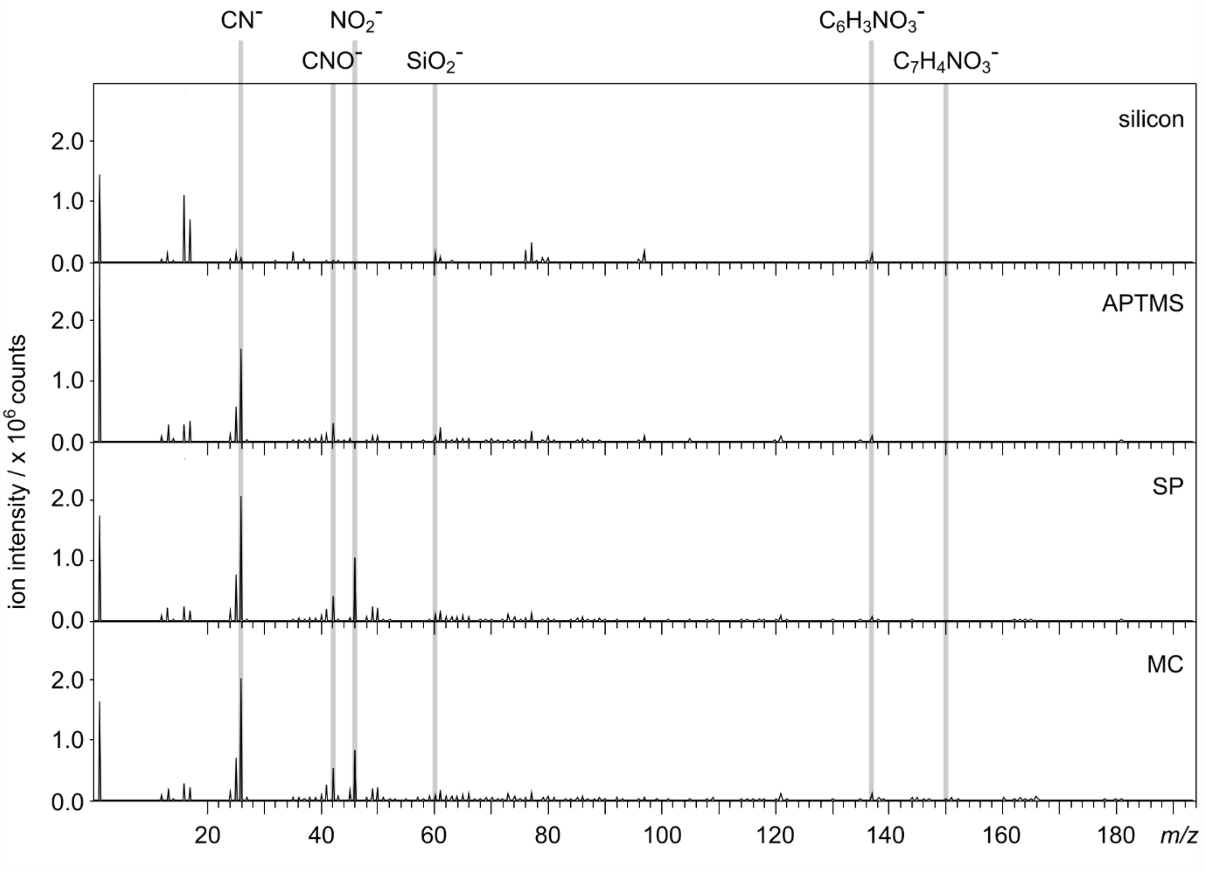

Figure S5. Surface analysis of the modified surfaces by ToF-SIMS. Negative ion spectra in the lower m/z region for silicon, the APTMS modified surface (APTMS), the spiropyran surface (SP) and the SP surface after exposure of UV light to convert spiropyran to the merocyanine form (MC). Marker fragments used to distinguish between samples are highlighted.

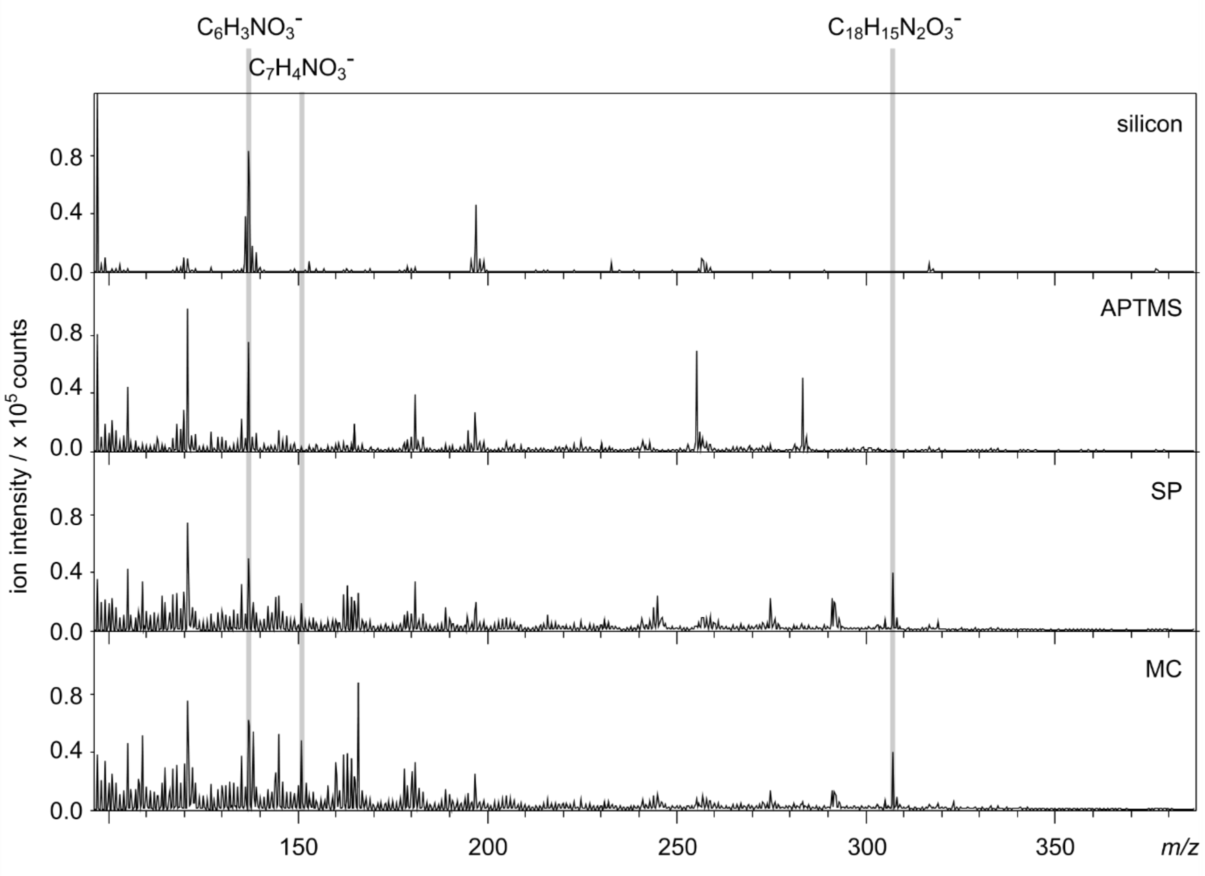

Figure S6. Surface analysis of the modified surfaces by ToF-SIMS. Negative ion spectra in the higher m/z region for silicon, the APTMS modified surface (APTMS), the spiropyran surface (SP) and the SP surface after exposure to UV light to convert spiropyran to the merocyanine form (MC). Marker fragments used to distinguish between samples are highlighted.

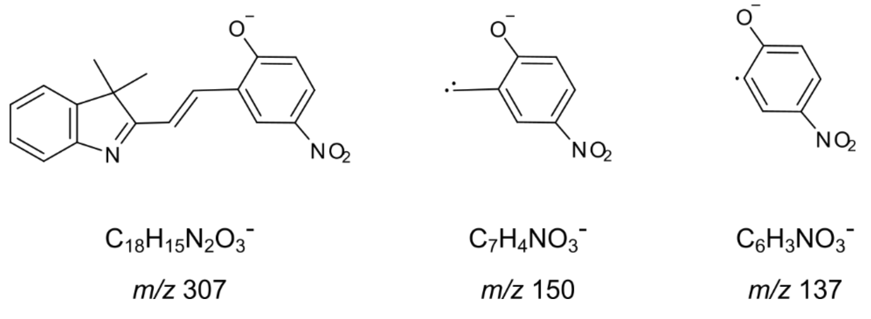

Figure S7. Chemical structures assigned to the SP and/or MC component of the SP-COOH surface that were identified in the ToF-SIMS spectra.

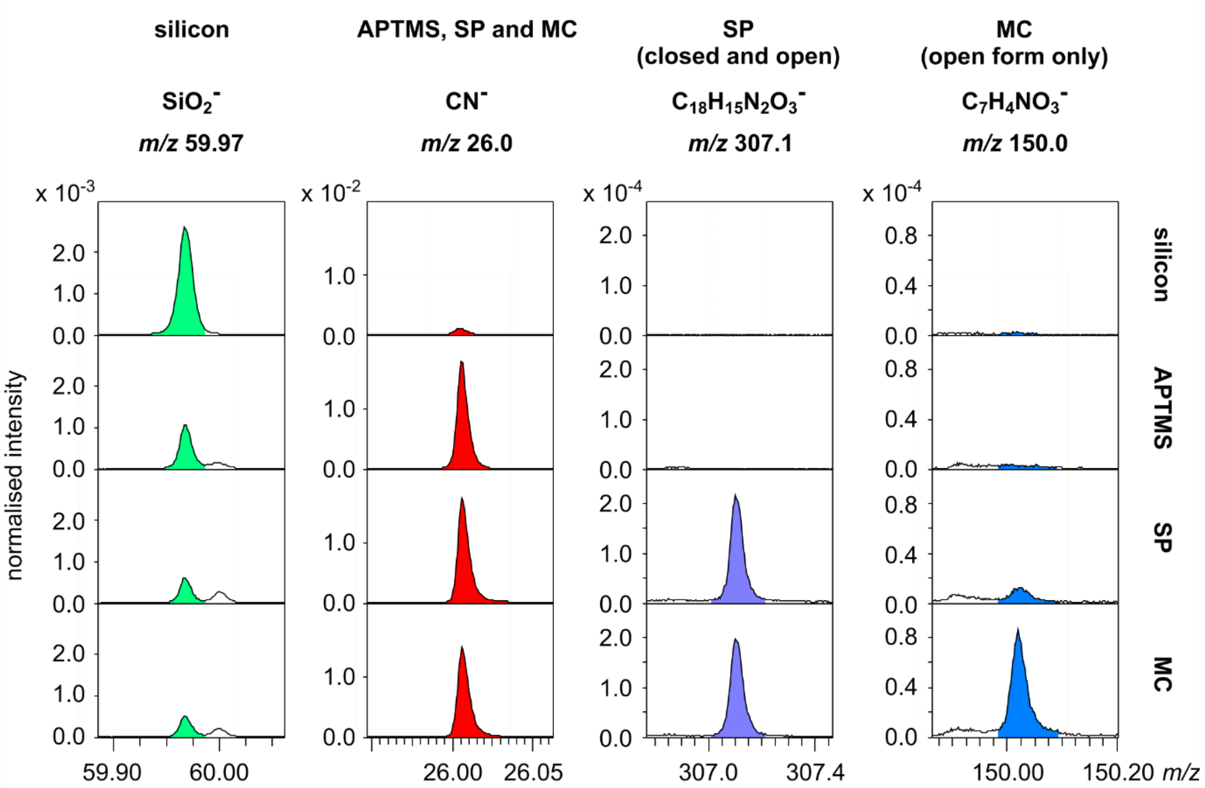

Figure S8. Relative intensities of marker ion fragments indicative for the different components on the modified surfaces identified in the ToF-SIMS spectra. Ions were normalised to the total ion intensity. For the MC form, the $C_7H_4NO_3^-$ ion at *m/z* = 150.0 was chosen over $C_6H_3NO_3^-$ at *m/z* = 137.0 due its higher intensity.

5. Photo-induced surface switching

SP surfaces were irradiated with UV light (365 nm, 8 W) or white light (300 – 600 nm, 3 W) for 12 hours to drive the population of molecules on the surface to predominantly adapt either the spiropyran (SP) or merocyanine (MC) form.

After conditioning of the SP surface under UV light, the resulting MC surfaces showed a significant decrease in WCA (Figure S4). In the ToF-SIMS spectra the presence of the MC form was characterised by the appearance of ions associated with the chromene moiety of the molecule ($C_6H_3NO_3^-$, $C_7H_4NO_3^-$) that were not observed at high intensities on the SP surface (Figures S5, S6 and S8). The ion indicative for the whole SP-COOH molecule ($C_{18}H_{15}N_2O_3^-$) was present on both the SP and MC surfaces. This indicates that while the ion intensity of the molecular ion is not affected by the switching, the generation of smaller fragments is facilitated by the conversion of the ring structure into an open structure on the MC surface.

6. Morphological and mechanical properties of dry gel films

AFM experiments were performed on a Bruker Dimension ICON with PeakForce QNM module installed. An AFM probe (Bruker RTESPA-150) with a spring constant of 6.77 N/m and a typical tip radius of 8 nm was used for the imaging of morphology and the measurement of mechanical properties in this study (Figure S9). The images in Figure S9 were processed in Gwydion 2.59; the polynomial background was removed (horizontal & vertical degree of 2), rows were aligned (method: matching) and horizontal scars were corrected before applying a filter (sharpen, size: 5 pixels). The gel was prepared as described in the main document on either the SP or MC surfaces and dried under ambient environment.

The deflection sensitivity of the tip was first calibrated on a clean silicon wafer. Then, various indentation depths of 20-400 nm were tested to rule out the substrate effects on the gel film mechanical properties. For the actual measurement, seven different locations across the whole sample were chosen to obtain the Young's modulus using the PeakForce QNM method. On each of the locations, the image was collected on a 2 μm × 2 μm area at a scan rate of 1.0 Hz. The resolution of the image was 512 × 512 pixels. 700 evenly distributed locations were chosen to perform an independent indentation test. Various indentation depths of 20 – 400 nm were tested to rule out any potential effects of the silicon wafer substrate on the measurement and a 200 nm indentation was chosen for the measurement.

In this mode, the AFM cantilever was controlled to oscillate at a certain frequency and perform a tapping mode scan on the sample surface. During each tapping cycle, the force and separation curves were recorded. The surface modulus at the indentation point was derived from the unloading curve by using the Derjaguin-Mueller-Toporov (DMT) fitting model:[3, 4]

$$F = \frac{4}{3} E_r \sqrt{R d^3} + F_{adh} \tag{1}$$

where *F* is the force on the tip, *R* is the tip radius, *d* is tip deflection, and F_{adh} is the adhesion force between tip and sample. In this equation, these variables are recorded during experiments, leaving the reduced modulus, E_r, as the only unknown variable, which can be calculated by fitting the unloading curve using a power function in NanoScope Analysis 1.7. With the knowledge of the reduced modulus (E_r), the tip modulus (E_{tip}) and Poisson's ratio (ν), the Young's modulus of the sample (E_{sample}) could be obtained after rearranging the following equation:

$$E_r = \left(\frac{1-v_{sample}^2}{E_{sample}} + \frac{1-v_{tip}^2}{E_{tip}}\right)^{-1} \tag{2}$$

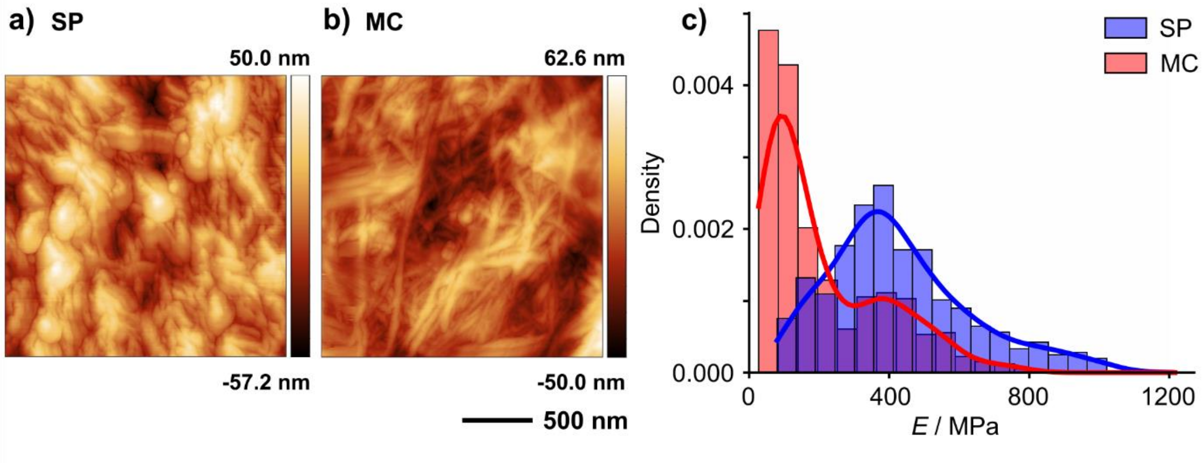

Figure S9. Morphology of dry gel on (a) SP and (b) MC surfaces visualised by AFM. Images have been processed to reduce background and line effects and sharpened using a filter. (c) The Young's modulus (*E*) of dry gels measured by AFM (tip radius = 8 nm).

7. Mechanical properties of uniform hydrated gel films

7.1. Quasi-static Nanoindentation Experiments

Quasi-static nanoindentation experiments were performed using a nanoindentation device (Chiaro, Optics 11) mounted on top of an inverted phase contrast microscope (Evos XL Core, Thermofisher). Measurements were performed at room temperature in air on hydrated hydrogel samples. For each sample, a set of single force-displacement (*F-z*) curves was acquired at a speed of 2 μm/s over a vertical range of 10 μm, changing the x point of every indentation by ~ 250 μm. The cantilever selected for the experiments had an elastic constant *k* of 0.34 N/m and held a spherical tip with a radius (*R*) of 22 μm, so that the mechanical properties of the samples are averaged over a reasonable area ($a = \sqrt{2R\delta}$, where a is the contact radius and δ is the indentation) (see Figure S10 a and b).

The forward segment of the collected curves was analysed using a custom software[5] programmed in Python 3 and the Numpy[6]/Scipy[7] scientific computing stack. Briefly, data were first aligned using a baseline detection method based on the histogram of the force signal. *F-z* curves were then filtered using a Savitzky Golay filter with a window length of 100 nm to remove random noise. After, *F-z* curves were converted to force-indentation (*F*-δ) curves by finding the contact points (z_0, F_0) using a thresholding algorithm, and computing the indentation according to:

$$\delta = (z - z_0) - (F - F_0) \quad (3)$$

To quantify the elastic properties of the gels, *F*-δ curves were then fitted with the Hertz model (Equation 4) up to an indentation of about 10% of the tip radius, and the Young's Modulus (*E*) extracted. The Poisson's ratio (ν) was taken as 0.5 assuming material's incompressibility given the highly hydrated nature of the gels.

$$F = \frac{4}{3}\frac{E\delta^{\frac{3}{2}}R^{\frac{1}{2}}}{(1-\nu^2)} \tag{4}$$

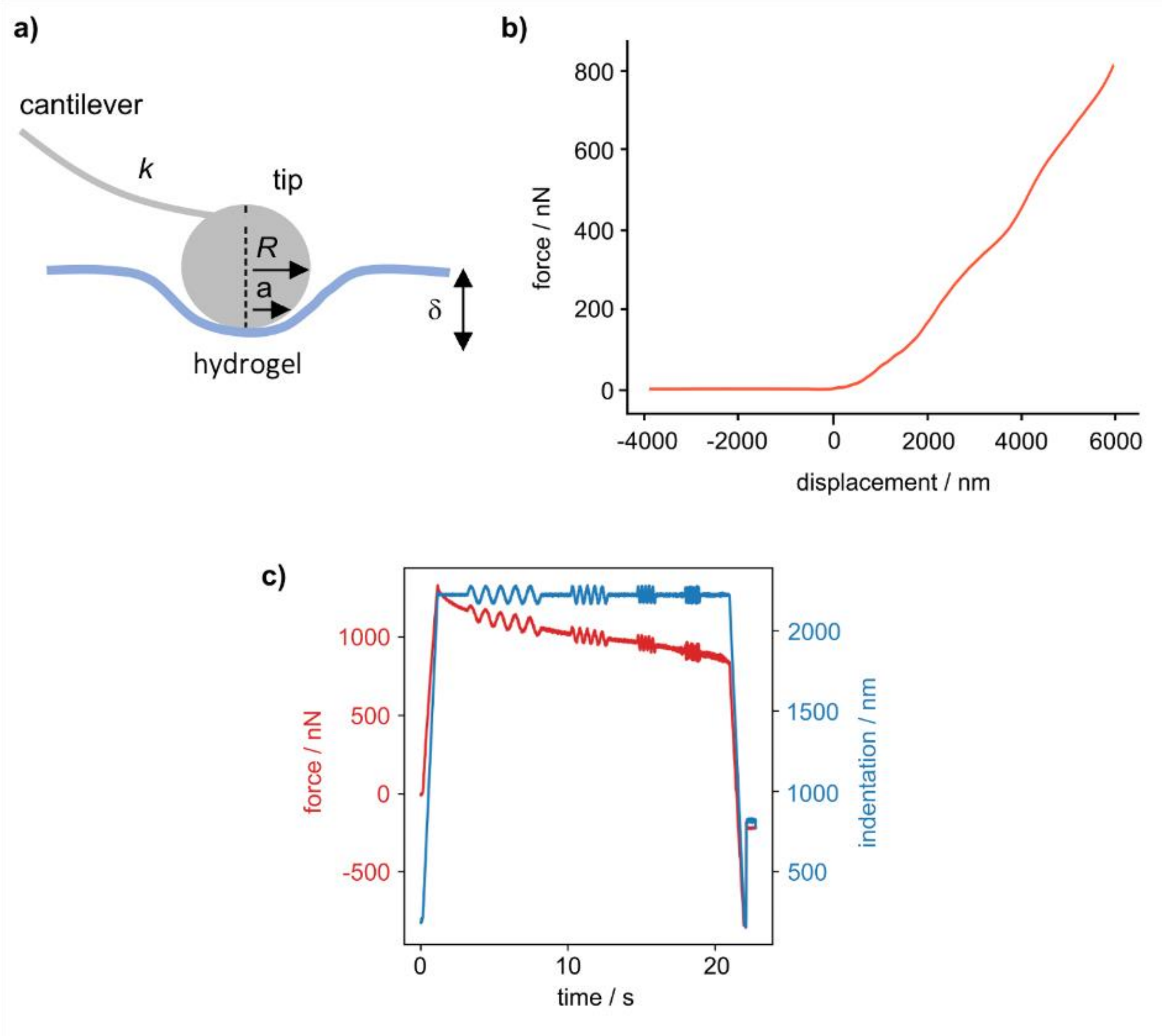

Figure S10. Mechanical characterisation of the hydrated 2-NapAV gels via nanoindentation. (a) Schematic of the nanoindentation process where a bead of radius *R* at the end of a cantilever with a spring constant *k* is used to indent the hydrogel by an amount δ, resulting in a contact radius *a*. (b) Representative *F-z* (force vs displacement) curve obtained on a hydrated 2-NapAV gel to characterise the gel's Young's Modulus. (c) Representative stress relaxation data with superimposed

dynamic oscillations obtained on a hydrated 2-NapAV gel to characterise the gel's storage and loss modulus.

7.2. Dynamic Nanoindentation Experiments

Dynamic nanoindentation experiments were performed using a nanoindentation device (Chiaro, Optics 11) mounted on top of an inverted phase contrast microscope (Evos XL Core, Thermofisher). Measurements were performed at room temperature in air on hydrated hydrogel samples. For each sample, a set of curves was acquired by performing two matrix scans in different regions of the gel. Each matrix scan consisted of 25 indentations, with a lateral step in x and y of 50 μm. Indentations consisted of stress relaxation experiments in dynamic mode, where a step indentation of 2000 μm with superimposed sinusoidal oscillations of amplitude 5 nm at 1,2,4 and 10 Hz was used as the input signal. The relaxing force was recorded over the time of the indentation, which was set to approximately 20 s (see Figure S10c).

The cantilever selected for the experiments had an elastic constant *k* of 0.34 N/m and held a spherical tip of radius (*R*) 22 μm in radius, so that the mechanical properties of the samples are averaged over a reasonable area ($a = \sqrt{2R\delta},$ where *a* is the contact radius) (see Figure S10a).

Data were analysed using the manufacturer's software (DataViewer V2.4, Optics 11). In brief, the sinusoidal oscillations were fit to a cosine function, and the estimated amplitude and phases were used to obtain the storage (E^{I}) and loss (E^{II}) moduli, which are a measure of the material's elasticity and viscosity, respectively:

$$E'(f) = \frac{(1-\nu^2)}{2a}\frac{F_0}{\delta_0}cos(\phi) \quad (5)$$

$$E''(f) = \frac{(1-\nu^2)}{2a}\frac{F_0}{\delta_0}sin(\phi) \quad (6)$$

Here, *f* is the frequency of oscillation; F_0 and δ_0 are the amplitudes of the oscillatory force and indentation signal, respectively; ϕ is the phase shift between the imposed oscillatory indentation signal and recorded force; ν is the Poisson's ratio, taken as 0.5 as previously described; and *a* is the contact radius, as previously defined. Since the contact area changes with the oscillatory signal, the contact radius is calculated using the average indentation depth given the small oscillatory amplitude of the indentation (5 nm).[8] G^{I} and G^{II} were obtained following *E=3G,* and all fits with an R^2 lower than 0.8 were rejected. The shear moduli at a frequency of 4 Hz were used for statistical comparison (see Figure 2 in the main text).

8. Characterisation of patterned surfaces

8.1. Preparation of patterned surfaces

Spatially defined conversion of an SP-COOH modified sample into either the SP or the MC surface was performed by selective irradiation of the two different areas on the same sample. Irradiation was carried out for at least 12 hours and performed simultaneously by controlling exposure to white (300 – 600 nm, 3 W) and UV (365 nm, 8 W, 0.458 mW cm^{-2} at sample position) light on the surface via optically sealed containers (Figure S11).

Gels were prepared on the surfaces immediately after patterning. 2-NapAV (2.5 mg) was suspended in deionized water (0.5 mL). An equimolar quantity of NaOH was added and the mixture was gently stirred for two hours until a clear solution was formed. The pH of this solution was 10.7. To prepare hydrogels, 2.32 mg glucono-δ-lactone (GdL) powder was added to tune the final pH to 4. The mixed 2-NapAV-GdL solution (350 µL) was then applied on a modified surface and left overnight in a sealed petri-dish to ensure complete gelation before the nanoindentation measurements.

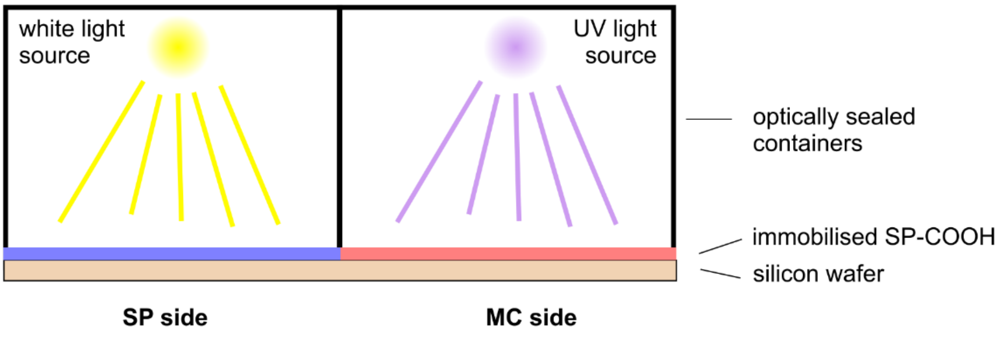

Figure S11. Schematic side view of the preparation of patterned samples. An SP-COOH modified silicon wafer was simultaneously exposed to white (300 – 600 nm, 3 W) and UV (365 nm, 8 W, 0.458 mW cm^{-2} at sample position) light for 12 hours. Selective irradiation of the surface was achieved by containing the radiation within optically sealed containers.

8.2. Chemical characterisation of surfaces by ToF-SIMS

The patterned sample (without gel) and the gel prepared on the patterned sample were analysed by ToF-SIMS to determine if the materials were chemically different on the SP and MC sides of the sample. The sample acquisition parameters were the same as those detailed above. The analysis area used (6 mm × 2 mm raster scans with 150 pixels × 50 pixels for SP-COOH surfaces and 6 mm x 1 mm with 150 pixels x 25 pixels for gel surfaces) spanned across the interfacial region of the SP and MC parts of the surface. For the patterned surface without gel, the same ions that were identified above were used to create ion maps that visualise the distribution of SP-COOH ($C_{18}H_{15}N_2O_3^-$; both closed and open form) and the open merocyanine form of the molecule ($C_7H_4NO_3^-$). On the sample containing the dried gel, an ion indicative of the gelator molecule ($C_{10}H_7O^-$ originating from the naphthyl group) was used to generate an ion image of the distribution of the gelator (Figure S12).

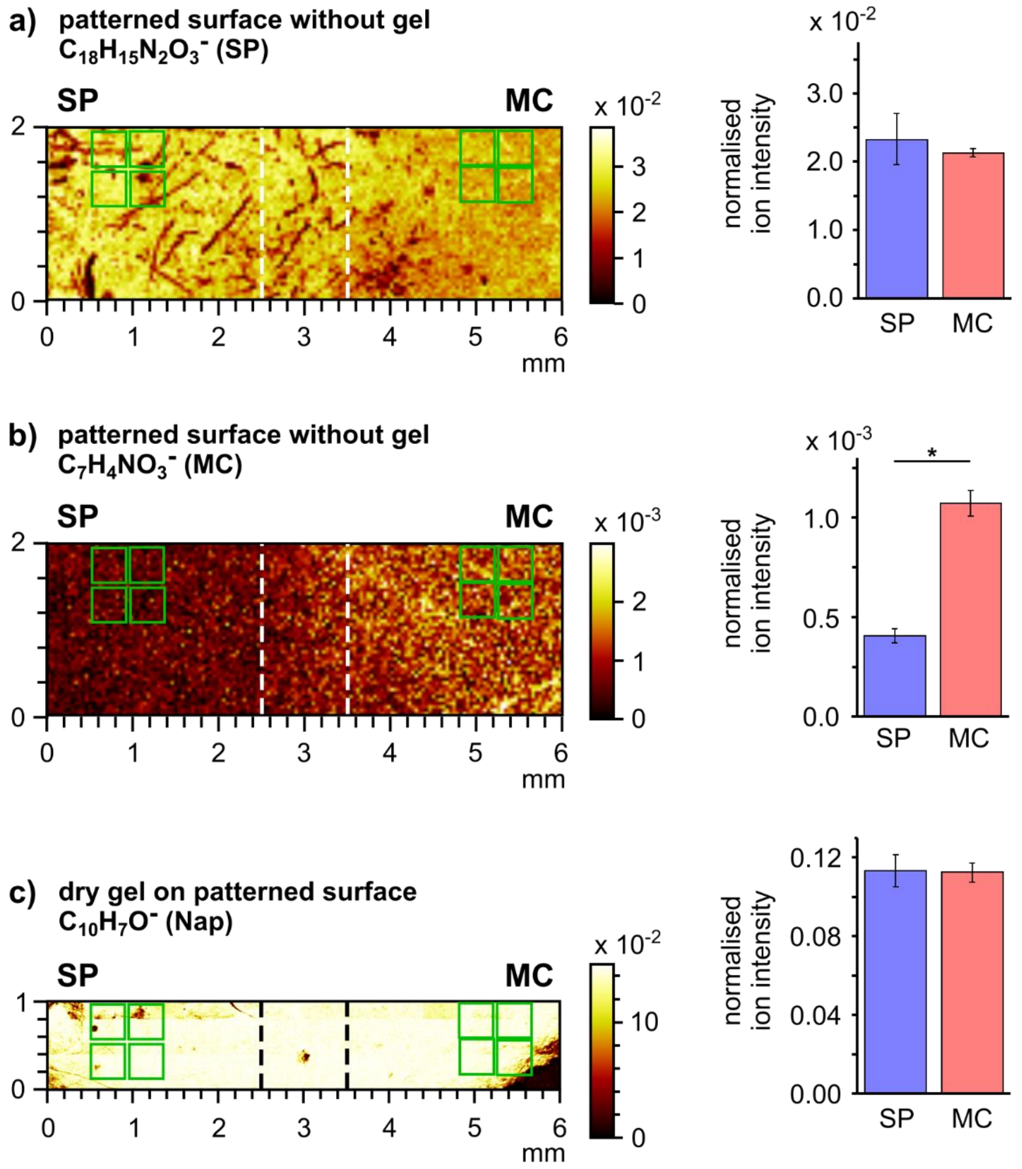

Figure S12. Distribution of ion intensities indicative of (a) the SP-COOH molecule ($C_{18}H_{15}N_2O_3^-$; both open and closed from) on the patterned surface without the gel, (b) the open merocyanine form of the SP-COOH molecule ($C_7H_4NO_3^-$) on the patterned surface without the gel and (c) the gelator, 2-NapAV ($C_{10}H_7O^-$) on the dried gel formed on the patterned SP-COOH surface. Ion intensities are normalised to total ion counts. The dashed lines indicate the transition region between the SP and the MC side. Green boxes indicate the regions of interest from which ion intensities were extracted. These ion intensities were used to generate the graphs on the right of the figure, showing the normalised ion intensities of the respective ion on the SP and MC sides of the samples. Values are reported as mean ± standard deviation; n =4; the * indicates statistically significant difference (2 sample t-test, DF = 3, $p = 2.576 \times 10^{-4}$).

For semi-quantitative analysis of the intensities of the selected ions, four regions of interest of identical size (400 μm × 400 μm) were created on the SP and MC side. The ion intensities for these four regions were used to calculate an average ion intensity for the 3 ions of interest on both sides of the surface and are shown in Figure S12. Only the $C_7H_4NO_3^-$ ion showed a statistically significant difference on the MC and SP sides, whereas the other two ions are similar on both regions. This indicates that the whole silicon wafer sample was covered with SP-COOH and confirms that after patterning, the UV exposed part of the surface (MC side) indeed changed towards the open form. The lack of a difference in the $C_{10}H_7O^-$ ion intensity on the dry gel samples indicates that the gel formed on the patterned surface is chemically homogenous despite the chemical differences on the patterned surface at the start of the gel formation process.

8.3. Mechanical characterisation of patterned hydrated gels by oscillatory nanoindentation

The mechanical properties of wet gel films on the patterned samples were measured by oscillatory nanoindentation. Tests were performed to determine the complex shear modulus of these gels using a KLA-Tencor Nanoindenter G200 with a DCM-II Head (CA, US). Measurements were conducted with a 100 μm flat punch indenter (Synton-MDP Ltd., Nidau, Switzerland) at room temperature (25 ^{o}C) at 110 Hz with a pre-compression of 5 μm. 30 indentations across the centre of the sample were made from the edge of one surface to the other, covering a distance of about 10.8 mm. The distance between each indent is 400 μm. The storage modulus (*G′*), the loss modulus (*G″*) and the loss factor *tan(δ)* (i.e., the ratio of *G″*/*G′*) were calculated for each indentation. After each indent, the tip was cleaned by indenting a piece of double-sided Scotch tape mounted on an adjacent sample puck before returning to the gel sample. Figure S13a shows the layout of the samples used for the nanoindentation measurements. The *G′* and *G″* values measured across the patterned sample are shown in Figure S13b and S13c.

Control measurements were performed in the same fashion on gel prepared on individual, non-patterned SP and MC surfaces. To do this, the full SP-COOH sample was exposed to either UV or visible light before gel formation to obtain a chemically uniform SP or MC surface. Nanoindentation measurements were performed across the samples as described for the gels on patterned samples; the results are shown in Figure S14. Mean *tan(δ)* values were determined (0.173 ± 0.040 and 0.252 ± 0.020 for SP and MC surfaces, respectively) and compared via a two sample t-test. The values were found to be statistically significantly different ($p = 7.14 \times 10^{-8}$) between the gels on the SP and MC surfaces. In addition, to rule out potential measurement artefacts, the data from the control samples was divided into two groups of equal size at the centre of the measured length range. When comparing the mean *tan(δ)* values from these two groups, no statistically significant difference was found (two sample t-test, p = 0.097 and 0.121 for SP and MC, respectively). This confirms that the difference between the SP and the MC sides on the patterned sample is real and not a measurement artefact.

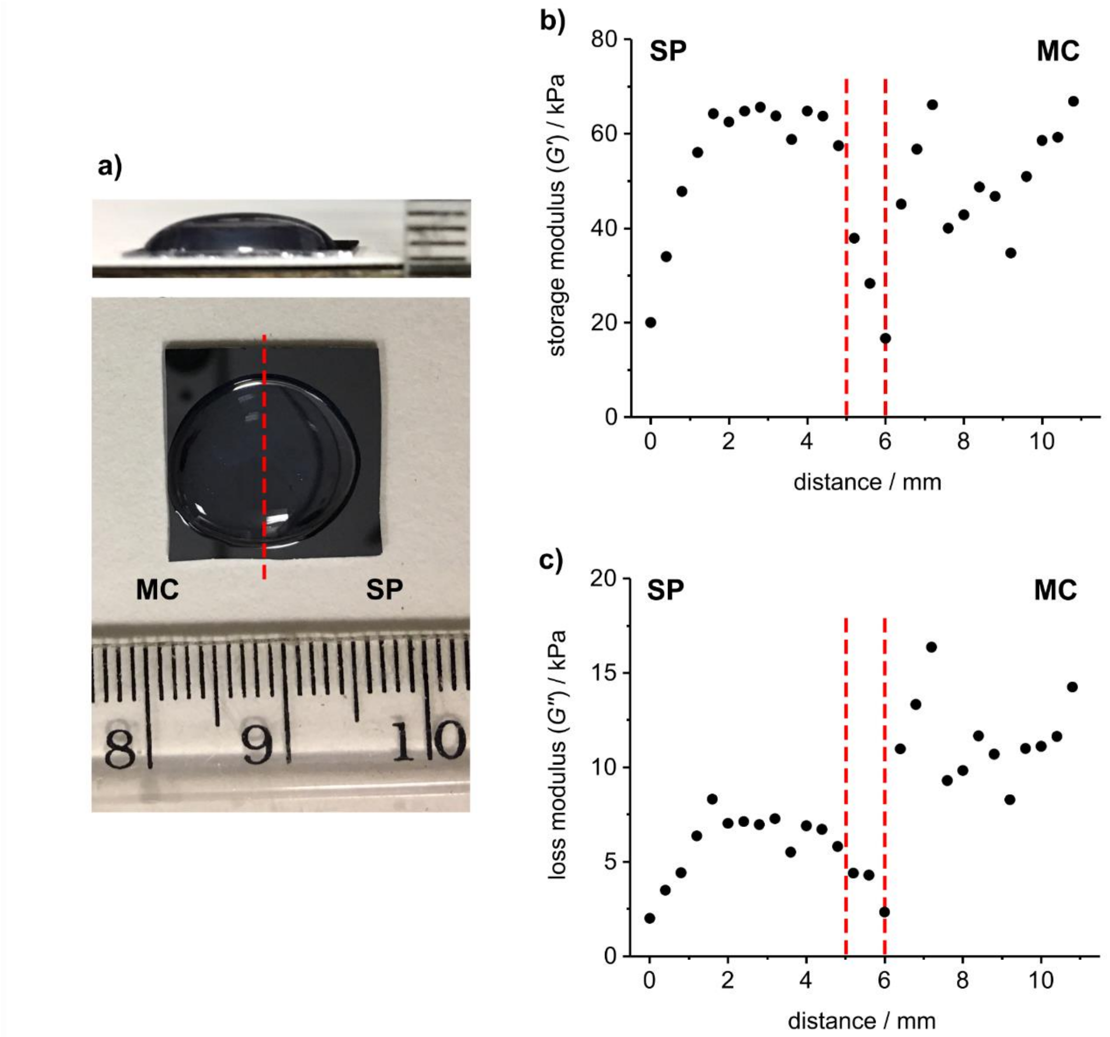

Figure S13. (a) Photograph of the wet gel prepared on the patterned surfaces for nanoindentation measurements and (b) the storage (*G′*) and (c) loss moduli (*G″*) obtained on the gel sample. The area between the red dashed lines is the transition area separating the SP and MC parts of the patterned surface during irradiation.

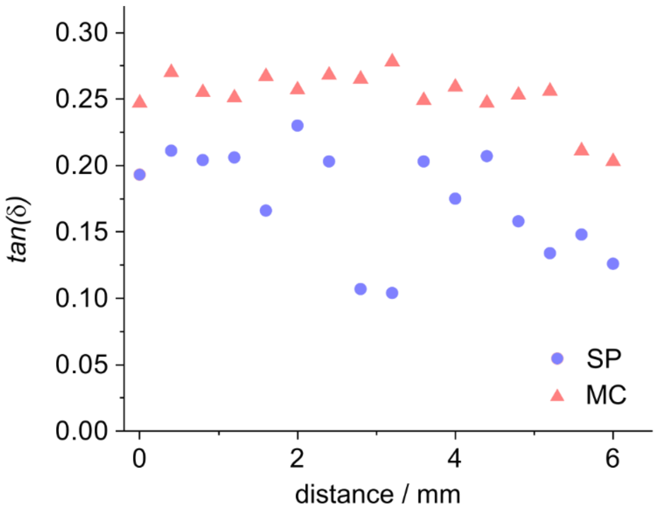

Figure S14. Loss factors (*tan(δ)*) obtained from gels formed on uniform SP and MC surfaces that were not patterned before gel formation.

9. Gel fibre structure

To evaluate the structure of the gel samples, grazing-incidence X-ray diffraction (GID) and grazing-incidence small-angle X-ray scattering (GISAXS) measurements were performed on gels formed on individual SP and MC surfaces. Details of the experimental procedure are provided in the main manuscript.

The sample was prepared using the method described in Section 5.1 on a SP-COOH treated silicon wafer. GID and GISAXS experiments were performed on the I07 beamline, Diamond Light Source, Didcot, UK. Time-resolved GID experiments were started immediately after the spreading of the hydrogel forming solution using X-rays with an energy of 18 keV, wavelength of 0.6888 Å to achieve a q range of 0.05 $Å^{-1}$-5.8 $Å^{-1}$. For time resolved GISAXS experiments, patterns with a q range of 0.027-0.6 $Å^{-1}$ were obtained using X-ray energy of 14.5 keV and a wavelength of 0.8551 Å. The sample-detector distance was 3 m.

9.1. Grazing-incidence X-ray diffraction (GID)

Grazing-incidence X-ray diffraction (GID) is used to probe the structure of the gel both parallel with the surface (along $q_{parallel}$) or perpendicular to the surface (along $q_{perpendicular}$). The diffraction patterns obtained for the GID experiments on gels on the SP and MC surfaces are shown in Figure S15. The images display some directional patterns. On the SP surface, the reflections at 0.55 $Å^{-1}$ and 1.34 $Å^{-1}$ are more dominant in the $q_{parallel}$ direction. On the MC surface, these two reflections are present in both the parallel and the perpendicular directions. On both surfaces, the position of these two peaks is slightly shifted in the patterns for the $q_{perpendicular}$ and $q_{parallel}$ directions compared to the patterns obtained when integrating over the whole diffraction image (Figure 4c and 4f). This is attributed to a small degree of orientation of the fibres that may have been introduced during the drying process. For the structural determination, the average values from the patterns obtained after full integration were used.

On the MC surface, the fully integrated spectra show new reflections at 0.09 $Å^{-1}$, 0.18 $Å^{-1}$ and 0.27 $Å^{-1}$ (*Q* ratio of 1:2:3) (Figure 4f). These reflections are only present in $q_{perpendicular}$, indicating that these peaks correspond to a directional order of the gel fibre bundles.

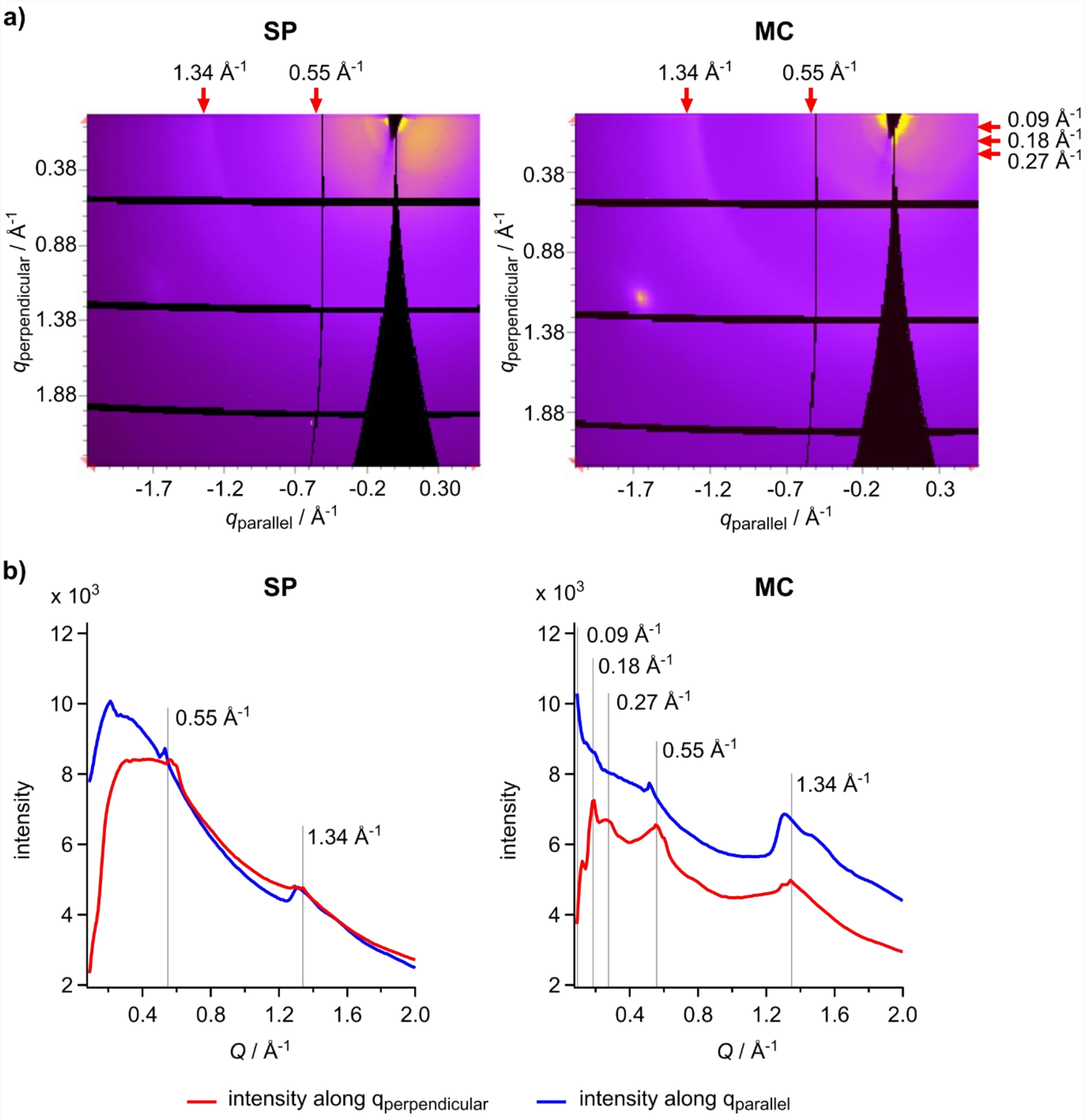

Figure S15. (a) 2D Grazing-incidence X-ray diffraction pattern (GID) of the 2-NapAV hydrogels formed on the SP and MC surfaces at 103 min and 76 min, respectively. The 2D images correspond to the patterns shown in Figure 4c. (b) Diffraction patterns obtained by integrating the 2D images in the parallel (blue) and perpendicular (red) direction. Annotations correspond to peak positions observed in the full integration of the 2D images and are intended for comparison with peak positions present in the parallel and perpendicular directions.

9.2. Grazing-incidence small-angle X-ray scattering (GISAXS)

While not strictly applicable to the grazing-incidence geometry, we can qualitatively analyse the small-angle X-ray scattering (SAXS) part of the data using standard bulk SAXS models. This is a useful indicative approximation of the full quantitative analysis. Thus, the data was fitted using a Kratky-Porod flexible cylinder model with polydisperse cross section and a uniform scattering length.[9, 10] This model describes a worm-like flexible chain of Length (*L*, the total length), made from freely jointed cylindrical units with a stiff segment length (*lp*), which is half of the Kuhn Length (*b*), and a cross-sectional radius of *R* (Figure S16). Polydispersity of the cross-section is included using a Schulz distribution. An overall scale factor that corresponds to the volume fraction of the cylinders is also included in the fitting parameters. For the fitting process, the restraining conditions were that the Kuhn length had to be larger than 2*R*, and smaller than the overall length *L*. The scattering length densities (*SLD*) used in the fitting were 10.470 × 10^{-6} Å^{-2} and 9.469 × 10^{-6} Å^{-2} for 2-NapAV and water, respectively. The fitting results are shown in Figure S17 and Table S1.

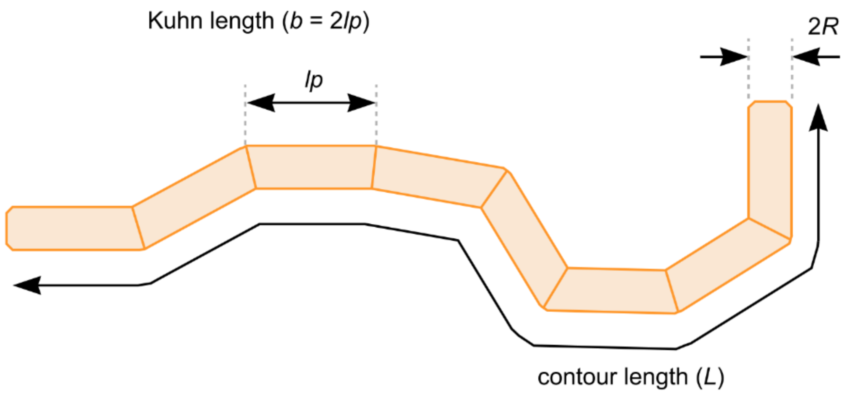

Figure S16. Schematic showing the parameters associated with the dimensions of the worm-like chain in the Kratky-Porod flexible cylinder model.[9, 10]

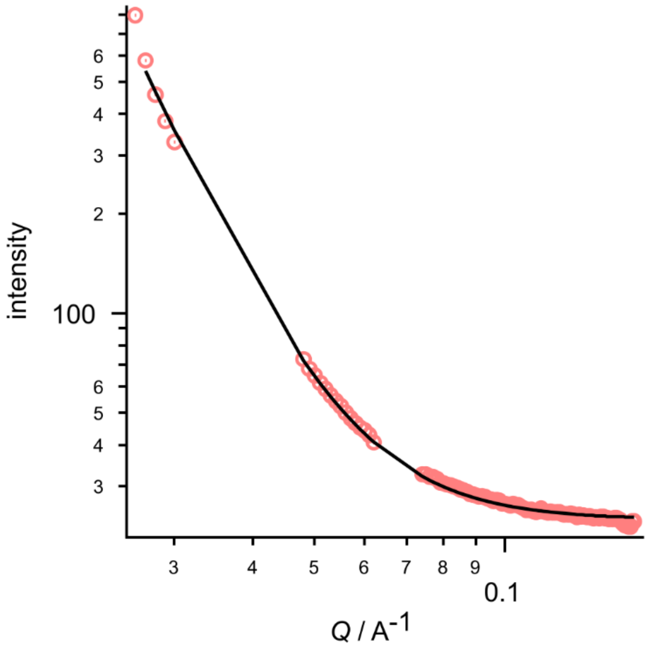

Figure S17. First GISAXS patterns of 2-NapAV wet gel on the surface of MC. The solid line on the pattern is a fit to the data with a Kratky-Porod flexible cylinder model. Data points between 0.030 $Å^{-1}$ - 0.048 $Å^{-1}$ and 0.062 $Å^{-1}$ - 0.074 $Å^{-1}$ are missing due to masking by the reflective beam-stop and the spaces between the detector plates, respectively.

Table S1. The model fit parameters generated by fitting the GISAXS pattern of gels formed on the surface of MC with a Kratky-Porod flexible cylinder model in the NIST SANS analysis package. The chi square value is 1.64.

Parameter	Fitted value
Contour Length, *L* (Å)	75900.4 ± 28130.5
Kuhn Length, *b* (Å)	165.4 ± 15.3
Radius, *R* (Å)	77.9 ± 0.3
Polydispersity of Radius	0.802 ± 0.004

10. Replicates and statistical analysis

Where averages are reported, all values are presented as mean ± standard deviation.

Water contact angle measurements were performed on two separate samples with a total number of repeat measurements of n = 5, 6, 6, and 4 for the silicon wafer, APTMS, SP and MC surfaces, respectively.

Peak force measurements were carried out on seven different locations on the SP and the MC surfaces. 700 measurements were performed at each location. All data from one surface was pooled and used to plot a frequency distribution of the occurrence of Young's moduli on each of the two surfaces.

ToF-SIMS data from non-patterned samples was collected from a 500 µm x 500 µm area with 256 × 256 pixels each to qualitatively confirm surface modification. Each pixel represents an individual mass spectrum. N = 1, 2, 3 and 3 for silicon, APTMS, SP and MC surfaces, respectively. One representative image has been used to generate the spectra shown.
For patterned surfaces, large area scans with a length of 6 - 8 mm across the interface and a height of 1.2 – 3.2 mm were acquired from three separate samples each for the SP-COOH and the gel surface. The dimensions varied to provide a sufficiently large area to ensure measurement of the interface and at the same time minimise the analysis time required. From these images, smaller areas of 6 mm x 2 mm (1200 pixels x 400 pixels) and 6 mm x 1 mm (1200 pixels x 200 pixels) were cropped from the SP-COOH and the gel surface samples, respectively. The pixels were binned to combine 64 measurements (i.e. 64 individual pixels) into one larger pixel, giving an image resolution of 150 pixels x 50 pixels and 150 pixels x 25 pixels for the SP-COOH and the gel surface, respectively. While this reduces the spatial resolution of the image, it increases the signal intensity for each resulting pixel and allows a clearer visualisation of the change in ion intensities on the surface.
The data obtained from one of the three samples was selected to generate representative spectra and images and to perform statistical analysis of ion intensities on the two sides of the patterned surfaces. Four regions of interest were created on each side of the patterned surface as described in section 5.2 and their intensities were used to report a mean ion intensity ± standard deviation (n = 4). For statistical comparison of the ion intensities on the two sides of the patterned surface a 2 sample t-test was used with a significance level of 0.05 and assuming equal variance. Statistically significant differences are indicated in the figures and the p-values and degrees of freedom (DF) are reported in the figure captions. For the not significantly different comparisons, DF = 3 and p-values were 0.414 and 0.681 for the ion intensities of $C_{18}H_{15}N_2O_3^-$ and $C_{10}H_7O^-$, respectively.

Nanoindentation on patterned surfaces was performed on three separate samples; one representative sample was used to generate the figures and perform a statistical comparison between the two sides of the patterned surface. n = 14 on the SP and n = 12 on the MC surface as two data points in the transition area were excluded for the statistical analysis. Non-patterned control samples were measured once each with 16 measurements on each sample. For statistical comparison, the data was either pooled into two groups of 8, separating the data points at the centre of the measured line, to compare two different sections of the control samples (DF = 14; p-value = 0.097 and 0.121 for the SP and MC surface, respectively). To compare the values between the SP and MC control samples, all 18 data points from each sample were used. In all cases, statistics was performed with a 2 sample t-test using a significance level of 0.05 and assuming equal variance. Degrees of freedom (DF) and p-values of significantly different datasets are reported in the caption of the corresponding figures.

GID and GISAXS data were collected from one sample each for the SP and MC surfaces.

11. References

1. H. Jo, N. Haberkorn, J.-A. Pan, M. Vakili, K. Nielsch and P. Theato, *Langmuir*, 2016, **32**, 6437-6444.
2. L. Chen, K. Morris, A. Laybourn, D. Elias, M. R. Hicks, A. Rodger, L. Serpell and D. J. Adams, *Langmuir*, 2010, **26**, 5232-5242.
3. B. V. Derjaguin, V. M. Muller and Y. P. Toporov, *Prog Surf Sci*, 1994, **45**, 131-143.
4. L. Morales-Rivas, A. Gonzalez-Orive, C. Garcia-Mateo, A. Hernandez-Creus, F. G. Caballero and L. Vazquez, *Sci Rep-Uk*, 2015, **5**.
5. M. Vassalli, G. Ciccone and I. Lüchtefeld, 2021, February 5, CellMechLab/nanoindentation: beta, v0.1.1, Zenodo, https://doi.org/10.5281/zenodo.4508646.
6. C. R. Harris, K. J. Millman, S. J. van der Walt, R. Gommers, P. Virtanen, D. Cournapeau, E. Wieser, J. Taylor, S. Berg, N. J. Smith, R. Kern, M. Picus, S. Hoyer, M. H. van Kerkwijk, M. Brett, A. Haldane, J. F. del Río, M. Wiebe, P. Peterson, P. Gérard-Marchant, K. Sheppard, T. Reddy, W. Weckesser, H. Abbasi, C. Gohlke and T. E. Oliphant, *Nature*, 2020, **585**, 357-362.
7. P. Virtanen, R. Gommers, T. E. Oliphant, M. Haberland, T. Reddy, D. Cournapeau, E. Burovski, P. Peterson, W. Weckesser, J. Bright, S. J. van der Walt, M. Brett, J. Wilson, K. J. Millman, N. Mayorov, A. R. J. Nelson, E. Jones, R. Kern, E. Larson, C. J. Carey, İ. Polat, Y. Feng, E. W. Moore, J. VanderPlas, D. Laxalde, J. Perktold, R. Cimrman, I. Henriksen, E. A. Quintero, C. R. Harris, A. M. Archibald, A. H. Ribeiro, F. Pedregosa, P. van Mulbregt, A. Vijaykumar, A. P. Bardelli, A. Rothberg, A. Hilboll, A. Kloeckner, A. Scopatz, A. Lee, A. Rokem, C. N. Woods, C. Fulton, C. Masson, C. Häggström, C. Fitzgerald, D. A. Nicholson, D. R. Hagen, D. V. Pasechnik, E. Olivetti, E. Martin, E. Wieser, F. Silva, F. Lenders, F. Wilhelm, G. Young, G. A. Price, G.-L. Ingold, G. E. Allen, G. R. Lee, H. Audren, I. Probst, J. P. Dietrich, J. Silterra, J. T. Webber, J. Slavič, J. Nothman, J. Buchner, J. Kulick, J. L. Schönberger, J. V. de Miranda Cardoso, J. Reimer, J. Harrington, J. L. C. Rodríguez, J. Nunez-Iglesias, J. Kuczynski, K. Tritz, M. Thoma, M. Newville, M. Kümmerer, M. Bolingbroke, M. Tartre, M. Pak, N. J. Smith, N. Nowaczyk, N. Shebanov, O. Pavlyk, P. A. Brodtkorb, P. Lee, R. T. McGibbon, R. Feldbauer, S. Lewis, S. Tygier, S. Sievert, S. Vigna, S. Peterson, S. More, T. Pudlik, T. Oshima, T. J. Pingel, T. P. Robitaille, T. Spura, T. R. Jones, T. Cera, T. Leslie, T. Zito, T. Krauss, U. Upadhyay, Y. O. Halchenko, Y. Vázquez-Baeza and C. SciPy, *Nature Methods*, 2020, **17**, 261-272.
8. N. Antonovaite, S. V. Beekmans, E. M. Hol, W. J. Wadman and D. Iannuzzi, *Sci. Rep.*, 2018, **8**, 12517.
9. J. S. Pedersen and P. Schurtenberger, *Macromolecules*, 1996, **29**, 7602-7612.
10. W. R. Chen, P. D. Butler and L. J. Magid, *Langmuir*, 2006, **22**, 6539-6548.
